# Nociceptor-localized KCC2 suppresses brachial plexus avulsion-induced neuropathic pain and related central sensitization

**DOI:** 10.1186/s13578-025-01354-5

**Published:** 2025-01-31

**Authors:** Hang Xian, Huan Guo, Yuan-Ying Liu, Sui-Bin Ma, Rui Zhao, Jian-Lei Zhang, Hang Zhang, Rou-Gang Xie, Xu-Cheng Guo, Jie Ren, Sheng-Xi Wu, Ceng Luo, Rui Cong

**Affiliations:** 1 Department of Orthopaedics, Xijing Hospital, Air Force Medical University, Xi’an, 710032 China; 2 Department of Neurobiology, School of Basic Medicine, Air Force Medical University, Xi’an, 710032 China

**Keywords:** Brachial plexus avulsion, Neuropathic pain, Central sensitization, Peripheral sensitization, KCC2, BDNF

## Abstract

Lack in understanding of the mechanism on brachial plexus avulsion (BPA)-induced neuropathic pain (NP) is the key factor restricting its treatment. In the current investigation, we focused on the nociceptor-localized K^+^-Cl^−^ cotransporter 2 (KCC2) to investigate its role in BPA-induced NP and related pain sensitization. A novel mice model of BPA on the middle trunk (C7) was established, and BPA mice showed a significant reduction in mechanical withdrawal threshold of the affected fore- and hind- paws without affecting the motor function through CatWalk Gait analysis. Decreased expression of KCC2 in dorsal root ganglion (DRG) was detected through Western blot and FISH technology after BPA. Overexpression of KCC2 in DRG could reverse the hyperexcitability of DRG neurons and alleviate the pain of BPA mice synchronously. Meanwhile, the calcium response signal of the affected SDH could be significantly reduced through above method using spinal cord fiber photometry. The synthesis and release of brain-derived neurotrophic factor (BDNF) was also proved reduction through overexpression of KCC2 in DRG, which indicates BDNF can also act as the downstream role in this pain state. As in human-derived tissues, we found decreased expression of KCC2 and increased expression of BDNF and TrκB in avulsed roots of BPA patients compared with normal human DRGs. Our results indicate that nociceptor-localized KCC2 can suppress BPA-induced NP, and peripheral sensitization can be regulated to reverse central sensitization by targeting KCC2 in DRG at the peripheral level through BDNF signaling. The consistent results in both humanity and rodents endow great potential to future transformation of clinical practice.

**Supplementary Information:**

The online version contains supplementary material available at 10.1186/s13578-025-01354-5.

## Introduction

Brachial plexus avulsion (BPA) is the most severe injury of the peripheral nervous system (PNS) involving the upper extremity [1]. Not only can it bring motor function loss of the affected limb, but also results in paresthesia of the innervation territory. Well-designed reconstruction surgical procedures can help restore satisfactory motor function after avulsion injury. However, the residual neuropathic pain (NP) is still an unsolved issue that needs special attention [2, 3]. If the pain is not effectively controlled, the following comorbidities will further worsen the quality of life of patients and their families. According to previous reports, the NP occurrence rate after BPA can reach up to 90% [4], while there’s still little known about the underlying mechanisms.

Injury on the somatosensory pathway, including the PNS level and the central nervous system (CNS) level, can induce severe pain, and the characteristic chronicity of this pain will further complicate the pathophysiology of the progress of the disease [5]. BPA is a kind of injury that involves both PNS and CNS, which is a different injury from others, such as SNI-induced NP [6] and CFA-induced inflammatory pain [7]. Accumulated evidence shows pain sensitization plays a crucial role in the development and maintenance of BPA-induced NP [8]. Targeting BDNF at the peripheral level can reverse the peripheral sensitization and related mechanical allodynia of BPA mice, and blocking the EZH2 in anterior cingulate cortex and the receptor of CCL2 in spinal dorsal horn (SDH) can also control the central sensitization and achieve an analgesic effect of BPA model [9–11]. An increasing number of evidence revealed dorsal root ganglion (DRG) at the peripheral level as the generator of pain [12–14], while, the afferent and efferent fibers have lost connection with the spinal cord once the BPA happens. Whether specific regulation of DRG neurons can synchronously control the activity of SDH and what is the domination role at the DRG to SDH loop level in BPA-induced pain is still unclear.

K^+^-Cl^−^ cotransporter 2 (KCC2) is a Cl^−^ extruder that uses the K^+^ gradient for maintaining a low concentration of intracellular Cl^−^, which is almost expressed in neurons [15, 16]. There are dynamic changes in the expression of KCC2 during maturation of CNS, and destruction of KCC2-mediated Cl^−^ transport will attenuate GABA_A_ receptor-mediated inhibition effect of signal transduction [15]. This phenomenon has also been identified in several pathological disorders, including neuropathic pain [17, 18]. Decreased KCC2 expression was observed in SDH tissue of NP mice and was proved to regulate pain state at the spinal cord level [19–21], and similar decreased level of KCC2 was also observed in inflammatory pain [22, 23]. KCC2 in higher levels of CNS, such as the medial prefrontal cortex (mPFC) and the hippocampus, has also been proven to play an important role in pain regulation [24, 25]. Upstream brain-derived neurotrophic factor (BDNF) and its high affinity receptor tropomyosin-related kinase B (TrkB) is the mostly identified possible cascade reaction that participates in the process of pain in CNS through regulating downstream KCC2 [17, 21, 26]. While, whether nociceptor-localized KCC2 at the peripheral level share a similar analgesic effect and its underlying molecular mechanism in BPA-induced pain are still poorly understood.

Here, we introduced a novel designed BPA mice model and systematically investigated the function plasticity change caused by KCC2 in DRG at the level of periphery, and further explored its effect on central sensitization through using spinal cord fiber photometry. Finally, to uncover the potential role of molecular target, the probable molecular mechanism focusing on KCC2 in both mouse and human DRG was also explored.

## Materials and methods

### Animals

Adult female C57BL/6 mice (20 ~ 30 g) were raised in constant temperature- and humidity-controlled environment with standard dark–light cycle (25 ℃, 12 h: 12 h), and were freely allowed access to food and water during the whole experimental protocols. All the animal procedures used in this study were approved by the Animal Care and Use Committee of the Air Force Medical University (IACUC No. 20220607).

### BPA modeling procedure

As we previously reported [9], mice were anesthetized through intraperitoneal (i. p.) injection of 1% pentobarbital sodium. A horizontal incision below the left clavicle was selected, and a deeper dissection was performed in order to expose the brachial plexus. The middle trunk (C7 root) of the brachial plexus was carefully dissected. A soft silk thread was used to hook the C7 nerve root and pull the thread with constant traction toward the distal limb to best mimic the avulsion mechanisms of root avulsion injury in clinic. Then, the DRG body of the C7 could be observed in the stump of the avulsed root. In the sham group, the brachial plexus was only exposed and dissected without any further lesion to the roots and nerves. Tissue layers and skin were finally sutured.

### Behavioral analysis

Mice were acclimatized to the testing room for at least 3 d before behavioral tests, and all mice were randomly assigned to different experimental groups. Individual test compartments for at least 1 h, and all the tests were finally completed by a same experimenter in blind manner.

#### Mechanical allodynia

Mechanical allodynia (Mechanical withdrawal threshold test) was tested through using a series of von Frey filaments ranging from 0.008 g to 2.0 g on an elevated metal mesh-bottomed platform (Danamic Globe, USA). Starting with 0.008 g, filaments were applied to stimulate the plantar surface of the left fore- and hind- paws. The ‘positive responses’ to the stimulation were recorded including rapid flicking and/or biting, licking and shaking of the paw while excluding walking movements. Each filament was applied for 10 times, and the positive paw respond frequency was recorded. The corresponding force required for a given filament to produce a 50% frequency of paw withdrawal is represented as the mechanical threshold.

#### Thermal hyperalgesia

Thermal hyperalgesia, also known as thermal withdrawal latency was tested through using infrared heating to the plantar surface of the left fore- and hind- paws of mice, and the corresponding response latency was read automatically by the device (IITC model 400, Woodland Hills, CA, USA). Mice were placed on a temperature-regulated glass platform heated to 30 °C, and the central plantar surface of the fore- and hind- paws was stimulated with a radiant heat source (50 W halogen bulb) directly through an aperture. The time elapsed between the onset of stimulation and paw withdrawal is recorded as paw thermal withdrawal latency. The fore- and hind- paws of each animal were tested 5 times, with a time interval of 5 min, and the average latency was calculated and analyzed. The maximum stimulation time is set to no longer than 20 s, in order to avoid tissue damage caused by long-term thermal stimulation.

#### CatWalk gait analysis

As described previously, Gait analysis has been proved as a reliable method for the assessment of pain-related behaviors and motor function of mice [9, 27, 28]. Briefly, mice were trained on the enclosed glass platform at least 3 times before gait analysis began. A high-speed camera preplaced under the platform that the mouse walked across voluntarily would capture the image of each footprint and transmitted the recorded data to gait analysis software (CatWalk XT, version 10.6; Noldus, Netherlands). Pain related parameters including max contact area (cm^2^), print area (cm^2^), print length (cm), print width (cm), and motor function related parameters including body speed (cm/s), step cycle (s), run duration (s), run average speed (cm/s) were finally analyzed to evaluate the pain degree and motor function of the affected paws.

### Western blot analysis

At 7 d after Sham or BPA operation, DRG tissues of mice were dissected from sham group (containing left C6-C8 DRGs) and BPA group (containing intact left C6 and C8 DRGs) and lysed by RIPA lysis buffer (50 mM Tris–HCl, pH 7.4, 150 mM NaCl, 5 mM EDTA, 1% Triton X-100, 0.5% sodium deoxycholate, 0.1% SDS) with protease inhibitors in it on ice. Protein concentration was measured by BCA Protein Assay Kit (Thermo scientific, USA) and mixed with 5 × loading buffer (CWBIO, China), and then heated at 98 ℃ for 10 min. Protein samples were loaded and separated by SDS-PAGE. Primary antibodies including rabbit anti-BDNF (1:1000, SAB, USA), goat anti-TrκB (1:800, R&D systems, USA), rabbit anti-KCC2 (1:1000, Milipore, USA), rabbit anti-β-actin (1:2000, Proteintech, USA) and rabbit anti-GAPDH (1:2000, Proteintech, USA) were incubated at 4 ℃ overnight. After immunoblotted with corresponding second antibodies at room temperature for 2 h, proteins were visualized with an enhanced chemiluminescence detection method. The captured images were quantified by using Image J software (V1.6, USA). Specific bands for each protein were normalized to its respective β-actin or GAPDH.

### Immunofluorescence labeling and quantitative analysis

Mice were anesthetized and transcardially perfused with saline (20 mL) followed by 4% paraformaldehyde (PFA, 20 mL) at 7 d after Sham or BPA operation. Left cervical DRGs as western blot above of each group were removed and post fixed in 4% PFA overnight and then protected in 30% sucrose at 4 ℃ until sinking to the bottom of the tube. In brief, sections were cut on a cryostat (12 μm in thickness) and immunostained with primary antibodies including rabbit anti-KCC2 (1:400, Novus, USA) and goat anti-TrκB (1:800, R&D systems, USA) at 4 ℃ overnight. Second antibodies including Alexa Fluor 594 (goat anti-rabbit IgG, 1:1000, Abcam, UK) and Alexa Fluor 488 (donkey anti-goat IgG, 1:1000, Abcam, UK) were incubated at room temperature for 4 h. The nuclei were stained by DAPI (1:2000). All images were captured with an Olympus confocal microscope (Olympus FV3000, Japan). Captured images with a 40 × magnification under this confocal microscope were processed using Image J, and quantification of mean fluorescence intensity was performed manually in at least 8 sections from 3 to 5 animals.

### Fluorescence in situ hybridization and cell counting

As previously reported [29], the oligonucleotides, containing forward primer, 5'-CTCAACAACCTGACGGACTG-3'; and reverse primer, 5'-GCACAACACCATTGGTTGCG-3', amplify a 474 bp fragment (89–563) in the coding region of mouse KCC2 (GenBank database AF332064) was cloned and riboprobes were synthesized by digoxigenin (DIG) labeling. Mice were anesthetized and perfused with 0.01 M diethylpyrocarbonate-treated phosphate-buffered saline (DEPC-PBS, pH 7.4), followed by 4% paraformaldehyde (PFA, 20 mL) at 7 d after Sham or BPA operation. Tissues were post fixed in 4% PFA overnight and then protected in 30% sucrose dissolved in DEPC-PBS at 4 ℃ until sinking to the bottom of the tube. Sections were cut on a cryostat (12 μm in thickness) in RNase free environment and then acetylated for 10 min and pre-incubated for 1 h at 60 °C and then hybridized for 18 h at 60 °C with 1 μg/mL sense or antisense DIG-labelled KCC2 riboprobes in hybridization buffer. Following hybridization, the sections were incubated in a mixture of peroxidase-conjugated anti-DIG antibody (1:100) and one of the following antibodies: mouse anti-CGRP (1:1000, Abcam, UK), isolectin B4 DyLight 594 (IB4 DyLight 594, 1:300, Vector Labs, USA) and mouse anti-NF200 (1:200, Sigma-Aldrich, USA) overnight at room temperature. The KCC2 probe hybridization signal was amplified by using the biotinylated tyramine-glucose oxidase. After washing with DEPC-PBS, the second antibody including Alexa Fluor 594 (donkey anti-mouse IgG, 1:1000, Abcam, UK) was incubated at room temperature for 4 h. All images were captured with an Olympus confocal microscope (Olympus FV3000, Japan). Cell counts were made manually in at least 5 sections from 2 to 3 animals. All counting experiments were conducted in blind manner to the experimental group.

### Injection of rLV carrying overexpression RNA into DRGs and medication delivery

As described previously [9], mice were anesthetized and placed on the standard brain stereoscopic locator (RWD, China). Left C6-C8 DRGs were exposed through a longitudinal incision on the back of the neck. The microsyringe with glass pipette was inserted into the ganglion to a depth of 100 ~ 150 μm after dissection of epineurium over cervical DRGs. Microprocessor-controlled minipump containing 700 nL of virus solution was used to deliver virus vector to C6, C7 and C8 DRGs at the constant rate of 70 nL/min. The pipette was removed from ganglion after a further delay of 10 min. The tissue layers were carefully sutured and mice were allowed to recover on a 37 ℃ warming blanket. Mice were allowed to recover for 1 w before following BPA modeling, behavioral analysis and DRG patch-clamp recording. rLV (synthetized by BrainVTA, Wuhan, China) carried KCC2 overexpression RNA (rLV-EF1α-KCC2-PGK-mcherry-WPRE, rLV-OE group) and control rLV (rLV-EF1α-mcherry-WPRE, rLV-Ctrl group) were used in this study protocol. CLP 290 (MCE, China), the agonist of KCC2 was intraperitoneal (i. p.) injected to mice with a dosage of 100 mg/Kg as previous reports [30, 31], and the same dose of vehicle was used in the control group. These antagonists started use on 7 d after BPA, and then once a day for 5 consecutive days. Then the following behavioral analysis was conducted (Figs. 4A, 5A). Mice were perfused as described above and the expression of virus was confirmed by immunofluorescence analysis. We also firstly delivered rAAV2/8-hSyn-EGFP-WPRE-PA to the avulsed left C7-DRG to confirm its innervation into the palm of ipsilateral forepaw, and both the DRG, fiber and skin of the corresponding limb was confirmed by fluorescence analysis.

### DRG patch‑clamp recording

As described previously [9], mice were anesthetized and the whole DRG of mice were removed from sham group (containing left C6-C8 DRGs) and BPA group (containing left C6 and C8 DRGs, similar in rAAV injection groups). The DRGs were incubated in trypsin solution for 15 min at 37 ℃ after dissection of epineurium under microscopy. Then, the prepared DRG tissue was incubated in the incubation solution at room temperature for following use. The incubation solution was saturated with a mixture of 95% O_2_ and 5% CO_2_ at 26 °C for at least 1 ~ 3 h before experiment. DRG tissue was transferred into the recording chamber and superfused with oxygenated recording solution at the rate of 2 mL/min at room temperature (HEKA Electronik, Germany). Whole-cell patch clamp recording was performed using glass pipette with a resistance of 3 to 5 MΩ that was filled with pipette solution. DRG neurons were visualized under a 40 × water-immersion microscope (BX51WI; Olympus, Tokyo, Japan) equipped with infrared differential interference contrast optics. Whole-cell current and voltage recordings were acquired with HEKA EPC-10 USB amplifier (HEKA Electronik, Germany) and PatchMaster software. Cells with clear outline were selected and recorded under high magnification microscope (Leica, Germany). Neurons carrying red fluorescence in the rLV groups were selected as target cells (Fig. 5F, G). The following parameters of the neurons under stable recording mode were recorded under certain stimulation intensity, including resting membrane potential (RMP), membrane resistance (Rm), membrane capacitance (Cm), access resistance (Ra) and hold value. Agonist of KCC2 CLP290 was used at the concentration of 10 μmol and 50 μmol in this protocol to test its effect on the cell excitability of the target neurons. Parameters were selected to evaluate the cell excitability (Clampfit 10.7, USA) and statistics were analyzed.

### Spinal cord fiber photometry

Mice were anesthetized and placed on the standard brain stereoscopic locator. Corresponding SDH of left C6-C8 DRGs were exposed through a longitudinal incision on the back of the neck. The microsyringe with glass pipette was inserted into the left SDH to a depth of 150 ~ 250 μm. Microprocessor-controlled minipump containing 700 nL of virus solution (rAAV2/9-CaMKIIa-GCaMP6s-EGFP-WPRE-pA) was used to deliver virus vector to SDH at the constant rate of 70 nL/min. The pipette was removed from SDH after a further delay of 10 min. A shaped stainless fixator with a hole in the middle was fixed on the cervical spine and the optic recording fiber with ceramic casing was carefully implanted (SFig 4 A-D). The tissue layers were carefully sutured and mice were allowed to revive. In a state of free-moving of mice, baseline the GCaMP6s fluorescence signals was recorded after the recovery for 21 d of virus injection and then BPA model was performed. 7 d after BPA modeling, the GCaMP6s fluorescence signals was recorded under the stimulation of pinch, pressure, brush, thermal and von Frey filaments (Fig. 6B). The stimulation modes above were selected to best simulate the stimulations we meet in our daily life. Captured calcium signals were bandpass filtered (MF525-39, Thorlabs, Inc., Newton, MA, United States), and converted in to a voltage signal through an amplifier. The voltage signals were then recorded by the multichannel fiber recording system (ThinkerTech, Fig. 6A). For data analysis [32], fluorescence change (ΔF/F) was represented by (F-F_0_)/F_0_. F_0_ represents the mean of the fluorescence values in the baseline period, while F represents the fluorescence values of each time point under stimulation. Corresponding periods were defined as −2 to 10 s during the stimulation period. The ΔF/F values of mice in each group were then quantified (MATLAB R2018, USA) and analyzed (GraphPad Prisim 9.5, USA). Mice with incorrect fiber location were excluded during the analysis. Mice were perfused as described above and the expression of virus was confirmed by immunofluorescence analysis.

### Analysis of BDNF secretion in presynaptic terminals of nociceptors with BDNF-pHluorin

rAAV vector carrying an expression of BDNF tagged with a pH-sensitive fluorescent protein (BDNF-pHluorin, rAAV2/8-hSyn-BDNF-Flag-pHluorin-WPRE-bGH pA) were delivered similarly as above to the corresponding left C6-C8 DRGs of cervical spine. After the recovery from 21 d of virus injection, mice were perfused as described above (Fig. 7E). Similar to previously reported [33], dorsal root-attached spinal slices were harvested and the super-resolution confocal microscopy (Olympus FV3000, Japan) was used to capture the activity-induced BDNF secretion which was labeled by changes in the fluorescence intensity of BDNF-pH puncta in the superficial lamina of SDH. Corresponding periods were defined as −30 to 170 s during the stimulation period. The fluorescence change of the interested region was quantified (CellSens software, Olympus, Japan) and analyzed (GraphPad Prisim 9.5, USA).

### Clinical materials and DRG tissues of human

BPA patients often suffered from severe neuropathic pain. Clinical material including gender, age, affected limb, time to first admission, avulsion roots, VAS scale of pain and etiology of BPA patients with severe pain were collected. Corresponding items of normal control human volunteers were also collected for the following analysis. These BPA patients who admitted to our hospital were in search of further reconstruction surgery treatment. Human DRG tissues from BPA patients were harvested during the procedure of partial resection of the avulsed nerve roots (including DRG cell bodies) and the following nerve anastomosis for the recovery of the function of the affected limb. The inclusion criteria include adult patients (18 ~ 60 years old) who suffered from chronic pain longer than 3 months after BPA. The collected avulsed nerve roots (including DRG cell bodies) of brachial plexus were used for further biochemical tests (western blot analysis and immunofluorescence labeling, similar as above described). Goat anti-TrκB (1:800, R&D systems, USA) and corresponding secondary antibody Alexa Fluor 488 (donkey anti-goat IgG, 1:1000, Abcam, UK) was used in this branch experiment. The current investigation was approved by the Ethics Committee of Xijing Hospital, the Air Force Medical University (No. KY20222228-F-1). Patients involved in this study have signed the informed consent form, and all specimens were handled in an anonymized way according to ethical and legal standards.

### Data analysis and statistics

All data were graphed in GraphPad Prism 9.5 and all data analysis were performed through using GraphPad Prism 9.5 and SPSS 20.0 software. Data are presented as the mean ± standard error of the mean, S.E.M. Data that met Tukey’s and Bonferroni’s tests were analyzed through using the two-tailed, unpaired, or paired *t*-test, one-way ANOVA, or repeated-measures ANOVA followed by Tukey’s multiple-comparison test. Unless otherwise specified, the *P* values shown in figures and texts are derived from the analysis of variance. *P* < 0.05 was considered as significant difference.

## Results

### BPA induces severe pain in human

**Fig. 1:**
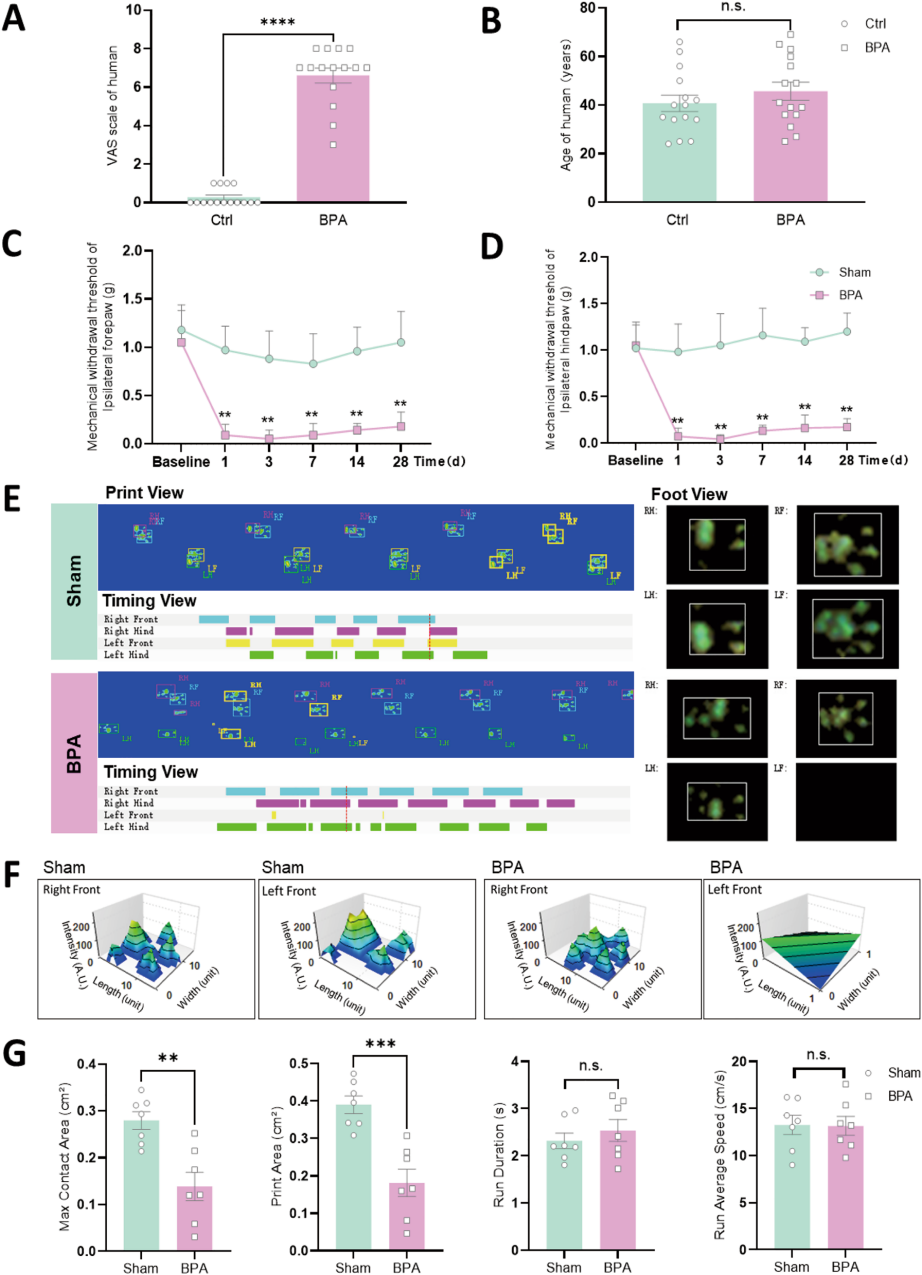
BPA induces severe pain in human and persistent mechanical allodynia of mice without affecting the motor function. **A** The VAS scale of BPA patients is higher than normal control human. **B** There’s no differences between BPA patients and normal control human in age. **C** Mechanical allodynia of the ipsilateral forepaw of BPA mice shows a decreased threshold on 1 d and lasts until 28 d, n = 8. **D** Mechanical allodynia of the ipsilateral hindpaw of BPA mice shows similar decreased of threshold. **E** Representative images of CatWalk gait including print view, timing view, and foot view of Sham and BPA models. **F** Three-dimensional reconstruction graphs of right and left forepaws according CatWalk gait analysis. **G** BPA induces a significant decrease in the max contact area and print area without affecting run duration and run average speed. Data are presented as the mean ± S.E.M. ^**^P < 0.01, ^***^P < 0.001, ^****^P < 0.0001, *vs* Sham or Ctrl. *n.s.* no significant difference, *RF* right forepaw, *RH* right hindpaw, *LF* left forepaw, *LH* left hindpaw

From January 2022 to January 2024, 15 BPA patients with NP were retrospectively reviewed. These include 14 males and 1 female with a mean age of 45.67 years. 7 cases with right upper limbs affected and 8 cases with left ones. The mean time interval between injury to admission is 2.73 months. There were 7 cases suffered by total avulsion of brachial plexus (C6-T1 roots), 4 cases of upper to middle trunk avulsion (C5, C6 and C7 roots) and 4 cases of upper trunk avulsion (C5 and C6 roots), and the avulsion injury were mostly caused by traffic accident. The mean VAS scale of preoperative pain is 6.6 (ranges from 3 to 8). The detailed clinical parameters of BPA patients refer to Table 1. We also collected the corresponding items of normal human volunteers, including gender (14 males and 1 female), age (mean 40.73 years) and VAS scale (mean 0.27). There exists significant difference in VAS scale between BPA patients and normal control human (Ctrl group), regardless of age (Fig. 1A, B). These results further confirmed that BPA could induce severe pain in human. While, this kind of pain is insensitive to the current treatment, searching for effective targeting treatment is still in urgent need.

**Table 1:** Clinical parameters of BPA patients

No	Gender	Age (years)	Left/right affected	Time to first admission (m)	Avulsion roots	VAS scale	Etiology
1	Male	60	R	4	C5, C6, C7, C8, T1	8	Traffic accident
2	Male	39	R	1	C5, C6	5	Traffic accident
3	Male	36	L	2	C5, C6	7	Fall injury
4	Male	56	R	1	C5, C6, C7, C8, T1	7	Fall injury
5	Male	41	L	3	C5, C6, C7, C8, T1	8	Strike injury
6	Male	69	L	1	C5, C6	4	Traffic accident
7	Male	49	L	2	C5, C6, C7, C8, T1	7	Traffic accident
8	Male	36	R	2	C5, C6, C7	7	Traffic accident
9	Male	39	L	4	C5, C6, C7	7	Traffic accident
10	Male	63	L	3	C5, C6	8	Fall injury
11	Male	25	R	2	C5, C6, C7	7	Strike injury
12	Male	65	R	5	C5, C6, C7	3	Fall injury
13	Male	31	L	7	C5, C6, C7, C8, T1	8	Traffic accident
14	Male	49	R	3	C5, C6, C7, C8, T1	6	Crush injury
15	Female	27	L	1	C5, C6, C7, C8, T1	7	Traffic accident
Mean	–	45.67 ± 13.77	–	2.73 ± 1.65	–	6.6 ± 1.45	–

### BPA induces lasting mechanical allodynia without affecting the motor function of mice

Following single 7th nerve root avulsion of brachial plexus, the mice showed persistent mechanical allodynia of the affected ipsilateral forepaw, and the onset of the pain state could last until 28 d after avulsion injury (Fig. 1C). We consider this kind of direct pain state evaluation is more reliable than other BPA models (reviewed by Xian et al. [4]). Meanwhile, the unaffected ipsilateral hindpaw showed a similar decrease in mechanical withdraw threshold like forepaw, which could be considered as pain sensitization at the CNS level (Fig. 1D). However, both the ipsilateral forepaw and hindpaw didn’t show thermal hyperalgesia after BPA (SFig 2A, B). To further confirm the reliability of direct pain detection of the affected forepaw, we introduced gait analysis to assess the pain behavior and motor function of the affected forepaw. Pain-related parameters, including max contact area, print area, print length and print width decreased after BPA, while, motor function related parameters including run duration, run average speed, body speed and step cycle showed no difference between sham and BPA groups (Fig. 1E–G, SFig 2C). The tracking results of virus carrying green fluorescence (EGFP) further confirmed the innervation of 7th nerve fiber into the affected forepaw (SFig 1A-C), which supplied the evidence on the connection of avulsed roots and the detected palm. These results indicated that BPA could induce lasting mechanical allodynia in the mice and single 7th nerve root avulsion won’t affect the motor function of the affected forepaw. Furthermore, both direct detection of affected forepaw (direct pain evaluation) and ipsilateral hindpaw (central sensitization evaluation or indirect pain evaluation) could be achieved through using this novel designed BPA mice model, which could be regarded as a more suitable BPA model as previously reported ones in this field. These results also proved that the unique characteristics of this novel BPA model could meet the requirements of the subsequent investigations.

### BPA injury decreases the level of KCC2 both in mice and human DRGs

**Fig. 2:**
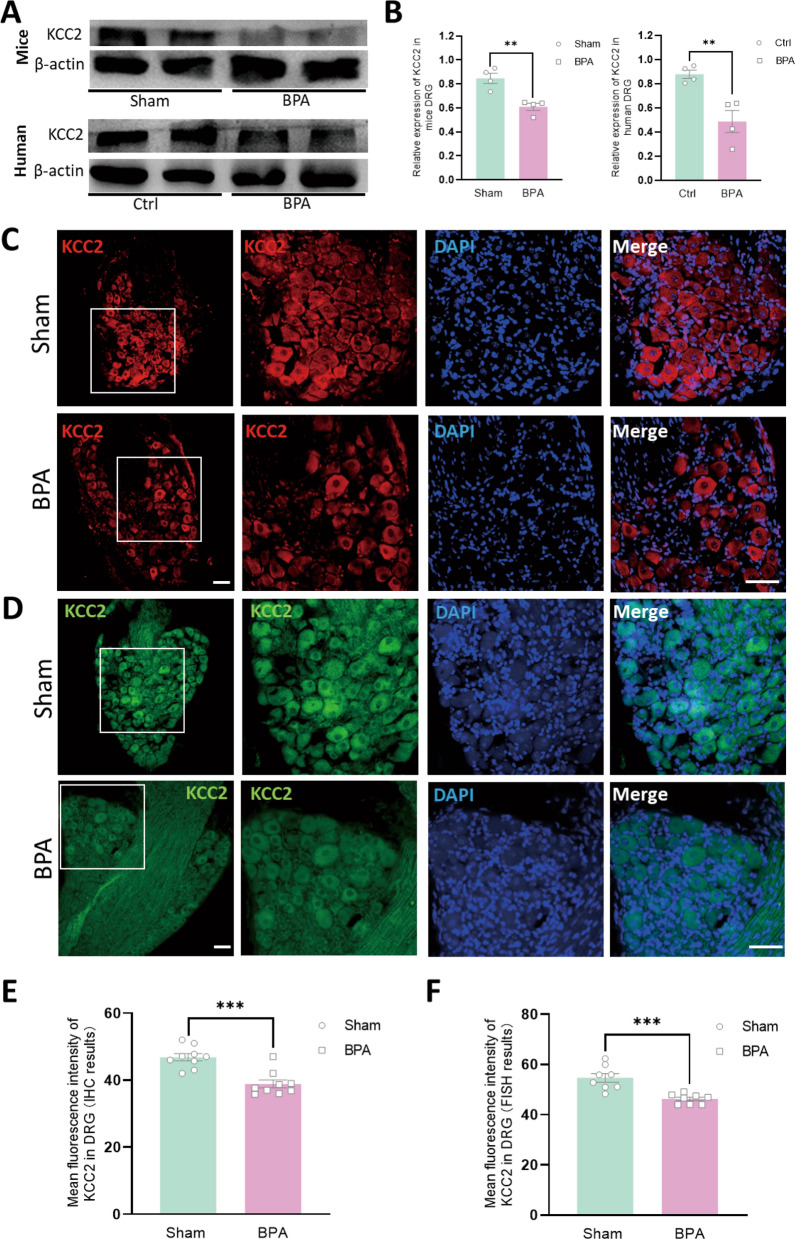
BPA induces decreased expression of KCC2 in ipsilateral DRGs of mice and BPA patients. **A** Typical examples of western blots showing the expression of KCC2 in the DRG tissue of the Sham and BPA mice, and in the DRG tissue of normal human and BPA patients. **B** BPA decreased the expression of KCC2 in the DRGs of mice and also in human. Data are normalized to the housekeeping protein β-actin. **C** Representative confocal images of KCC2 (red) in the DRG tissue of Sham and BPA mice using immunofluorescence labeling. **D** Representative confocal images of KCC2 (green) in the DRG tissue of sham and BPA mice using FISH staining. **E** Lower fluorescence intensity of KCC2 is observed in the BPA group using immunofluorescence labeling. **F** Lower fluorescence intensity of KCC2 is observed in the BPA group using FISH staining. Data are presented as the mean ± S.E.M. ^**^P < 0.01, ^***^P < 0.001, *vs* Sham or Ctrl. Scale bars, 50 μm

**Fig. 3:**
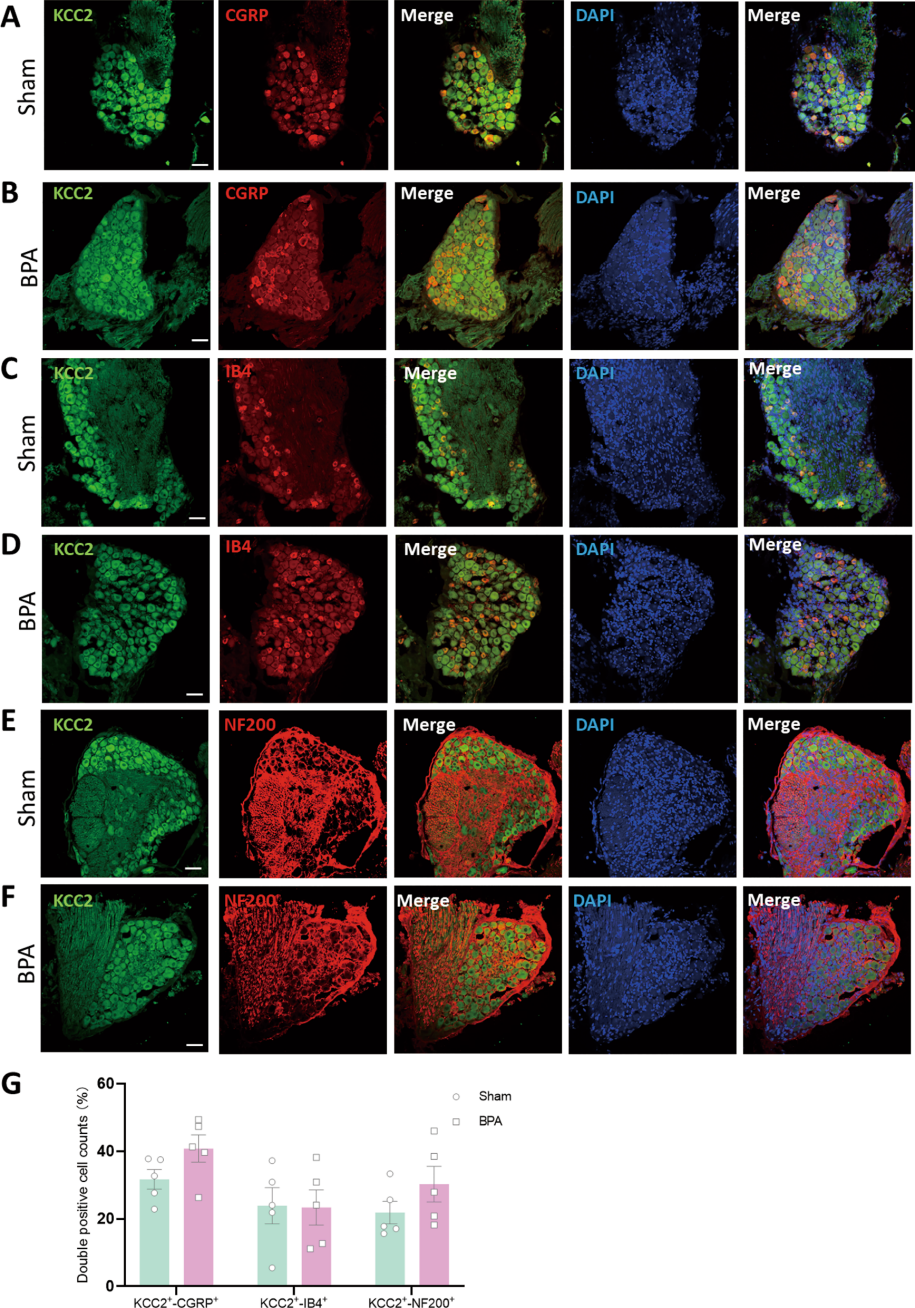
KCC2 in DRG shows no cell distribution difference through using FISH staining. **A** Representative confocal images of KCC2 (green) and CGRP (red) in the DRG tissue of Sham mice. **B** Representative confocal images of KCC2 (green) and CGRP (red) in the DRG tissue of BPA mice. **C** Representative confocal images of KCC2 (green) and IB4 (red) in the DRG tissue of Sham mice. **D** Representative confocal images of KCC2 (green) and IB4 (red) in the DRG tissue of BPA mice. **E** Representative confocal images of KCC2 (green) and NF200 (red) in the DRG tissue of sham mice. **F** Representative confocal images of KCC2 (green) and NF200 (red) in the DRG tissue of BPA mice. **G** Double positive cell counts show no difference in cell type distribution of KCC2 in DRG. Data are presented as the mean ± S.E.M. Scale bars, 50 μm

The expression profile and function of KCC2 have been well studied at CNS level [15, 31], while its function in peripheral level remains elusive. The expression of KCC2 decreased at 7 d after BPA through western blot test compared with sham ones, and similar decreased expression profile was also observed in DRGs of BPA patients compared with normal human DRGs (Fig. 2A, B). The results of immunofluorescence labeling also showed decrease of fluorescence intensity in mice compared with sham group (Fig. 2C, E). On considering the debate of the expression of KCC2 in DRG tissues, we further introduced fluorescence in situ hybridization (FISH) technology to examine the expression of KCC2 in DRG at transcriptional level. The results were consistent with the above western blot and immunofluorescence labeling (Fig. 2D, F). The cell distribution characteristics of KCC2 have also been assessed in FISH through co-staining with CGRP, IB4 and NF200, and the results showed there was no difference in the distribution of KCC2 in DRG neurons (Fig. 3A–G). Thus, the confirmation expression and decreased change of KCC2 in DRG induced by BPA might play a crucial role in the pathophysiological process of pain at the peripheral level, both in mice and clinical BPA patients.

### Regulation of KCC2 relieves pain and reduces the hyperexcitation of nociceptive DRG neurons induced by BPA

**Fig. 4:**
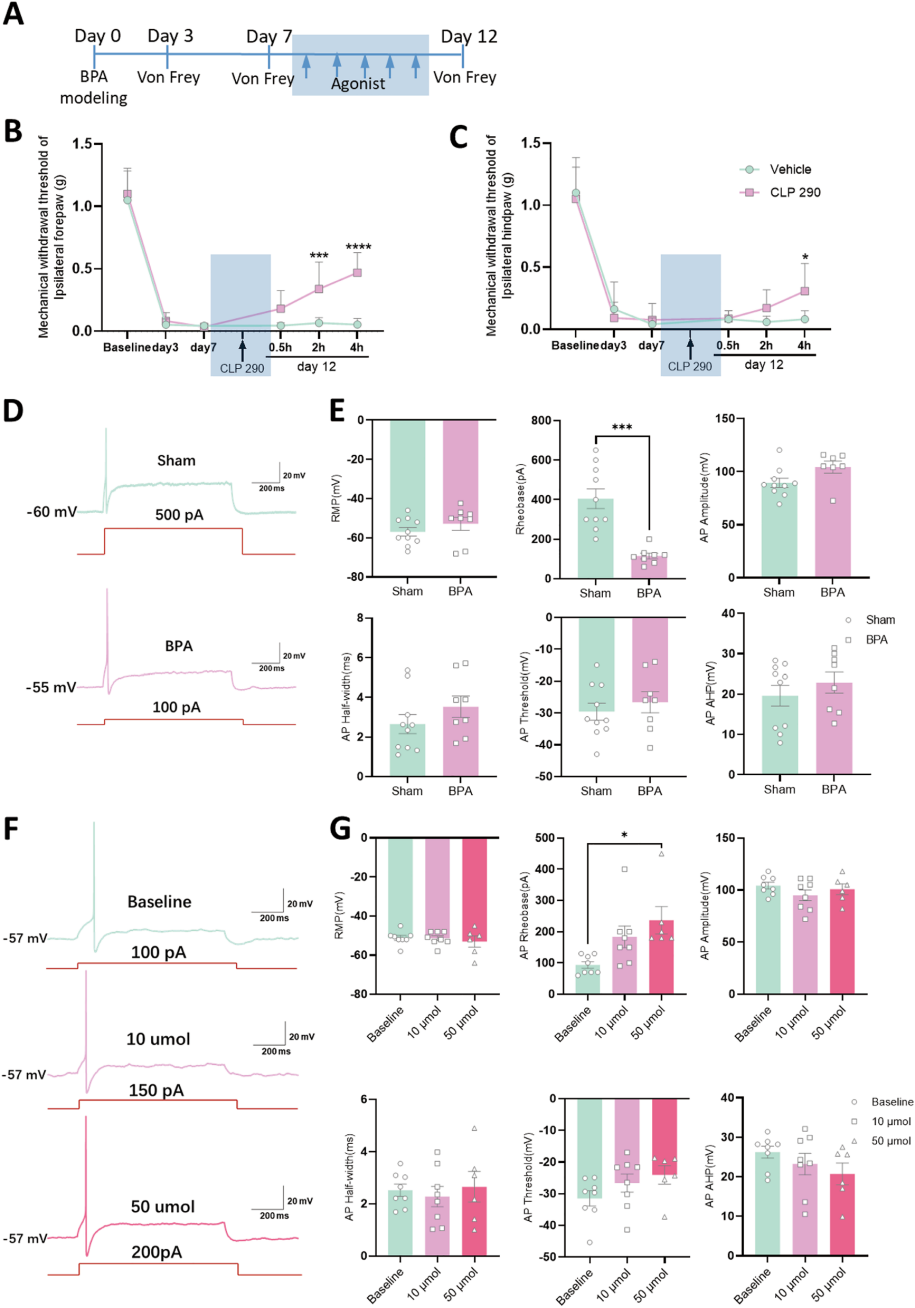
Agonist of KCC2 relieves pain and reduces the hyperexcitaion of nociceptive DRG neurons induced by BPA. **A** Schematic showing the agonist delivery groups and following tests. Agonist is started on 7 d after BPA, and then once a day for 5 consecutive days. **B-C** Mechanical allodynia threshold of the ipsilateral fore- and hind- paws is increased by i.p. injection of CLP290. **D** Representative traces of AP in the Sham and BPA groups. Neurons in the Sham group need stronger stimulation (500 pA) for firing. **E** BPA decreases the rheobase of DRG neurons after BPA but has little effect on the resting membrane potential (RMP), AP amplitude, AP half-width, AP threshold and AP after hyperpolarization potential (AHP). **F** Representative traces of AP in the agonist groups. Neurons in the higher concentration groups (10 μmol, 50 μmol) need stronger stimulation (150 pA, 200 pA) for firing. **G** Agonist at the concentration of 50 μmol increases the rheobase of DRG neurons, but has little effect on the RMP, AP amplitude, AP half-width, AP threshold and AHP. Data are presented as the mean ± S.E.M. ^*^P < 0.05, ^***^P < 0.001, ^****^P < 0.0001 *vs* Vehicle or Sham or Baseline

To explore the analgesic effect of KCC2, we first used the specific agonist of KCC2 (CLP 290). After 5 consecutive days of i.p. injection of CLP 290, the mechanical allodynia of the affected forepaw in BPA mice could be alleviated for at least 4 h (Fig. 4B), and mechanical allodynia of the ipsilateral hindpaw got relieved at the similar time (Fig. 4C). After this, we introduced whole cell patch‑clamp recording. Since whole cell patch‑clamp recording is the classical method for recording the excitability of excitable cells, which can supply direct evidence to the excitation changes of target cells. In DRG, medium to small diameter neurons govern the transmission of noxious stimuli, and the current patch‑clamp recording targeted these cell populations. The results showed the rheobase of the target neurons decreased dramatically compared with sham ones, and there’s no difference in RMP, AP amplitude, AP half-width, AP threshold and AP after hyperpolarization potential (AP AHP). Meanwhile, a lower stimulus intensity (100 pA) to neurons in BPA group could induce action potential (AP) compared with sham group neurons (500 pA, Fig. 4D, E). Patch‑clamp recording through using CLP290 solution perfusion showed the rheobase of the target neurons increased at the concentration of 50 μmol, and there also needs higher stimuli (200 pA) intensity to induce AP under higher concentration of agonist perfusion (Fig. 4F, G). These results proved the analgesic role of KCC2 in DRG neurons at the peripheral level.

### Targeting peripheral KCC2 can relieve the mechanical allodynia of mice

**Fig. 5:**
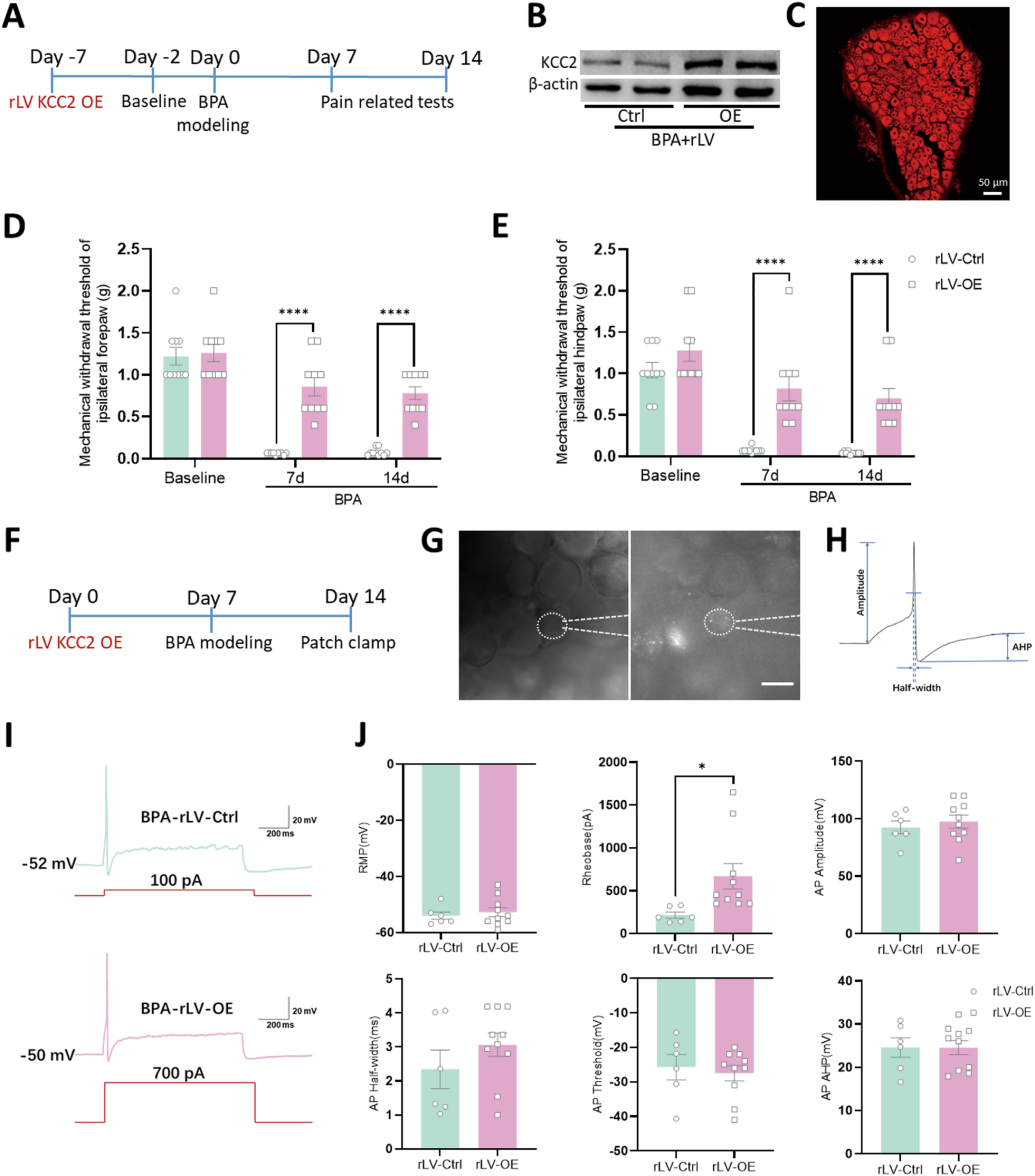
Up-regulation of KCC2 in DRG relieves the mechanical allodynia in BPA mice both in ipsilateral fore- and hind- paws and suppresses the hyperexcitation of nociceptive DRG neurons. **A** Schematic showing BPA modeling was performed on 7 d after virus delivery and pain behavioral test was performed on 7 d and 14 d after BPA. **B** Typical western blot images of KCC2 overexpression in the DRG tissue of mice. **C** Confocal image of KCC2 overexpression virus injected into the DRG (mcherry). **D**, **E** Overexpression of KCC2 in the DRG increases the mechanical withdraw threshold of ipsilateral fore- and hind- paws of BPA mice. **F** Schematic showing BPA modeling was performed on 7 d after virus delivery and patch clamp recording was performed on 7 d after BPA. **G** Representative images of the single-cell patch with fluorescence expression. **H** Representative trace of an action potential (AP) and marked analytical metrics. **I** Representative traces of AP in the rLV-Ctrl and rLV-OE groups after BPA. Neurons in the rLV-OE group need stronger stimulation (700 pA) for firing. **J** Up-regulation of KCC2 in DRG after BPA increases the rheobase of DRG neurons but has little effect on the resting membrane potential (RMP), AP amplitude, AP half-width, AP threshold and AP after hyperpolarization potential (AHP). Data are presented as the mean ± S.E.M. ^*^P < 0.05, ^****^P < 0.0001, *vs* rLV-Ctrl. Scale bar, 50 μm

In order to deeply mining the function of KCC2 during BPA-induced NP process, we delivered lentivirus vectors into ipsilateral DRGs to overexpress KCC2 (Fig. 5A). The confirmed expression of lentivirus was tested via western blot and fluorescence analysis (Fig. 5B, C). The pain behaviors including mechanical allodynia and thermal hyperalgesia have also been evaluated in both ipsilateral fore- and hind- paws to explore the analgesic role of KCC2. The mechanical withdraw threshold of paws increased in rLV-OE group compared with rLV-Ctrl group on 7 d and 14 d after BPA (Fig. 5D, E), but there’s no effect on thermal withdraw latency (SFig 3A, B). Patch‑clamp recording of the transfected neurons (carrying red fluorescence, Fig. 5G) showed an increase of rheobase and stimulus intensity (700 pA) to induce action potential compared with rLV-Ctrl group (Fig. 5I, J). The above results strongly indicate that the hyperexcitation of nociceptive DRG neurons induced by BPA can be suppressed through up-regulation of KCC2. Up to now, the analgesic effect of KCC2, especially at the peripheral level, has been confirmed. Here brings another question that peripheral manipulation of KCC2 could not only relieve the direct pain state of the affected forepaw (direct pain), but also could achieve pain relief of the ipsilateral hindpaw (central sensitization). Whether there is a close functional connection between DRG and SDH and the underlying molecular mechanisms under this phenomenon remain unclear.

### Overexpression of KCC2 in nociceptive DRG neurons alleviates centrally mediated secondary mechanical hypersensitivity

**Fig. 6:**
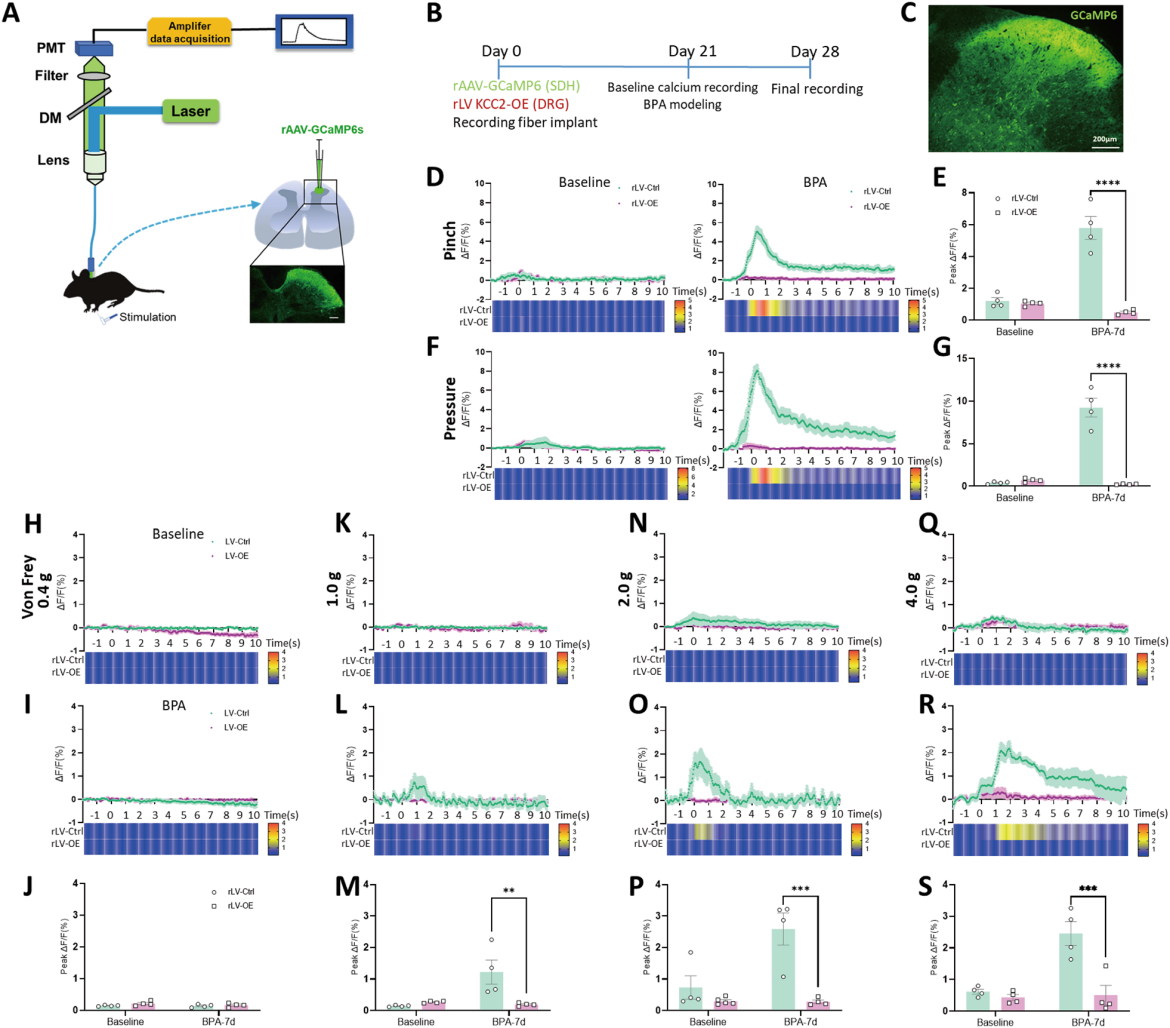
Manipulating KCC2 in DRG at the peripheral level suppresses calcium activity of neurons in corresponding SDH of cervical spine via stimulation to ipsilateral forepaw. **A** Schematic diagram of fiber photometry recording mode on cervical spinal cord. **B** Schematic showing the virus delivery method and following tests. BPA modeling started on 21 d and final recording started on 28 d after virus delivery. **C** Confocal image of virus carrying expression of GCaMP6s injected into the cervical SDH (EGFP). **D** Mean fluorescent signal of SDH under stimulation of pinch to ipsilateral forepaw was recorded, with shaded areas indicating the S.E.M. The red and green lines represent ΔF/F of rLV-Ctrl group and rLV-OE group, respectively. Heatmap represents above changes of GCaMP6s fluorescence intensity in these groups. The color scale at the right indicates ΔF/F. **E** Overexpression of KCC2 at the peripheral level suppresses calcium activity of neurons in SDH at the central level under the stimulation of pinch. **F** Similar parameters illustration under stimulation of pressure. **G** The calcium activity of neurons in SDH is also suppressed under stimulation of pressure. **H**–**J** Similar parameters illustration under stimulation of von Frey 0.4 g, and overexpression of KCC2 at the peripheral level has no different effect on calcium activity of neurons in SDH under this stimulation. **K**–**M** Similar parameters illustration under stimulation of von Frey 1.0 g, and the calcium activity of neurons in SDH at the central level can be suppressed under this stimulation. **N**–**P** Similar parameters illustration under stimulation of von Frey 2.0 g, and the calcium activity of neurons in SDH at the central level can be suppressed under this stimulation. **Q**–**S** Similar parameters illustration under stimulation of von Frey 4.0 g, and the calcium activity of neurons in SDH at the central level can be suppressed under this stimulation. Data are presented as the mean ± S.E.M. ^**^P < 0.01, ^***^P < 0.001, ^****^P < 0.0001, *vs* rLV-Ctrl. Scale bar, 200 μm

We then continued to address the functional impact of peripheral manipulation of KCC2 on lower CNS, mainly focusing on SDH. It is widely accepted that the afferent fiber from DRG neurons terminates in the superficial lamina of SDH, and neuroactive mediators that released from presynaptic terminals participate in the initiation and maintenance of pain. The activity of Ca^2+^ can be treated as a reliable index of neuron activities in the whole nervous system. Here, we creatively used fiber photometry of cervical spinal cord to explore the effect of peripheral KCC2 on SDH (DRG-SDH loop level). Surprisingly, the results showed calcium fluorescence intensity, marked by GCaMP6s, decreased obviously under the stimulation of ipsilateral forepaw including pinch, pressure (Fig. 6D–G) and von Frey (1.0 g, 2.0 g, 4.0 g, Fig. 6K–S) through peripheral overexpression of KCC2, and there’s little effect on brush, thermal stimulus (SFig 5A-D) and von Frey (0.4 g, Fig. 6H–J). The transfection effect of rAAV-GCaMP6s vector has been confirmed by fluorescence analysis (Fig. 6C). Under the stimulation of ipsilateral hindpaw, the calcium fluorescence intensity of the cervical SDH decreased obviously under the stimulus including pinch, pressure and von Frey (2.0 g, SFig 6A-D, 6G, H) through peripheral overexpression of KCC2, while there was no effect on thermal stimulus (SFig 6E, F). The results in this part indicate that neuron activities of CNS (SDH level) could be down regulated through a peripheral method, especially through overexpression of KCC2. This also answers the question why the direct pain of the affected forepaw (direct pain) and the ipsilateral hindpaw (central sensitization) could be relieved synchronously. In spite of this, the molecular mechanism relating KCC2 at this loop level is still far from being elucidated.

### Reinstating KCC2 expression in nociceptive DRG neurons reduces presynaptic BDNF synthesis and release in spinal terminals of nociceptors in turn

**Fig. 7:**
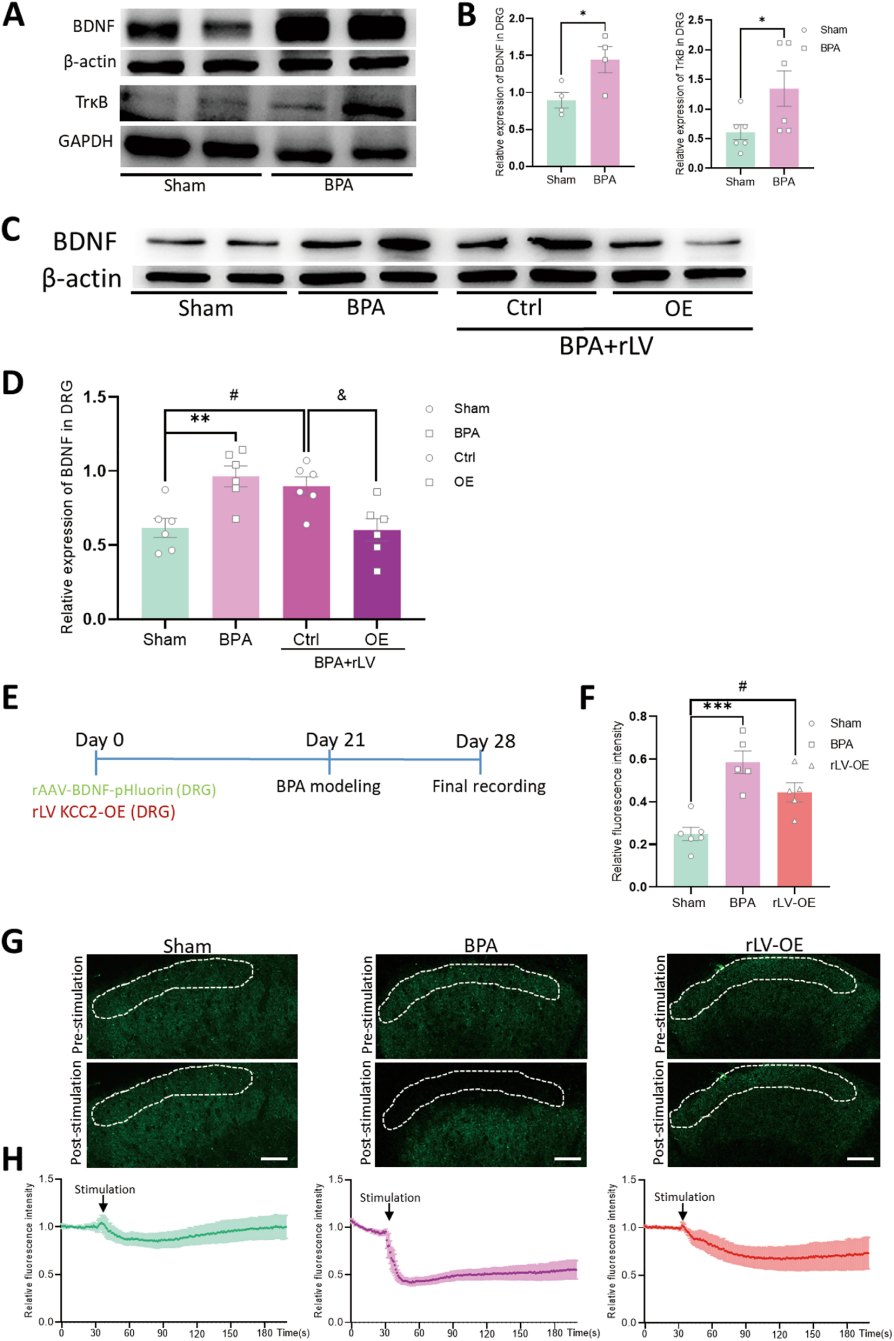
Presynaptic BDNF synthesis in DRG and release in SDH can be reduced through reinstating KCC2 expression in DRG neurons. **A** Typical examples of western blots showing the expression of BDNF and TrκB in the DRG tissue of the Sham and BPA mice. **B** BPA increased the expression of BDNF and TrκB in the DRGs of mice. Data are normalized to the housekeeping protein β-actin or GAPDH. **C** Typical examples of western blots showing the expression of BDNF in the DRG tissue after overexpression of KCC2. **D** Overexpression of KCC2 in DRG decreases the synthesis of BDNF in turn after BPA. **E** Schematic showing the virus delivery method and following tests. BPA modeling started on 21 d and final recording started on 28 d after virus delivery. **F** The relative BDNF-pHluorin fluorescence changes in SDH of BPA mice can be relieved through peripheral overexpression of KCC2. **G** Representative confocal images of BDNF-pHluorin fluorescence in SDH pre- and post-stimulation. Dotted portion represents superficial lamina of cervical SDH. **H** Mean fluorescent signal of BDNF-pHluorin fluorescence in SDH under stimulation was recorded, with shaded areas indicating the S.E.M. ^*^P < 0.05, ^**^P < 0.01, ^***^P < 0.001, *vs* Sham, ^#^*P* < 0.05, rLV-Ctrl/OE *vs* Sham, ^&^*P* < 0.05, rLV-Ctrl *vs* OE. Scale bar, 50 μm

BDNF and its high-affinity receptor TrκB are the mostly identified upstream signaling of KCC2 as reported in the CNS, which is known as BDNF-TrκB-KCC2 signaling. However, it remains uncertain whether a similar molecular cascade exists and plays arole in the peripheral DRG. Western blot tests of the corresponding DRGs of BPA mice showed increased expression of BDNF and TrκB on 7 d after BPA, which kept in line with previous reports in CNS (Fig. 7A, B). Further detection of BDNF revealed that high level expression of BDNF induced by BPA could be reduced by peripheral overexpression of KCC2 in DRG in turn (Fig. 7C, D). This resulting downregulation of BDNF maybe the possible molecular basis for the analgesic effect relating KCC2. That is, BDNF can also be the downstream molecular that could be regulated by KCC2 in DRG at the peripheral level.

To further search the evidence of peripheral dependent central sensitization, we expressed rAAV loaded BDNF tagged with a pH-sensitive fluorescent protein (superecliptic pHluorin; BDNF-pH) in the corresponding cervical DRGs. We observed high-level expression of BDNF-pH in spinal nociceptor terminals at 4 w after virus injection (Fig. 7G). The results showed that BPA could exacerbate the overall reduction magnitude of BDNF-pH fluorescence in SDH after stimulation, which could be mitigated through peripheral KCC2 overexpression (Fig. 7F–H). This demonstrates that the release manner of presynaptic BDNF in SDH is dependent on peripheral KCC2. These results infer a pivotal significance that peripheral BDNF synthesis and release depends on KCC2 signaling pathway in presynaptic plasticity during BPA-induced pain state. That is to say, KCC2 could also be the upstream regulator of previously reported upstream BDNF cascade at the peripheral level.

### Avulsed roots containing DRG neurons of BPA patients show similar molecular changes as BPA mice

**Fig. 8:**
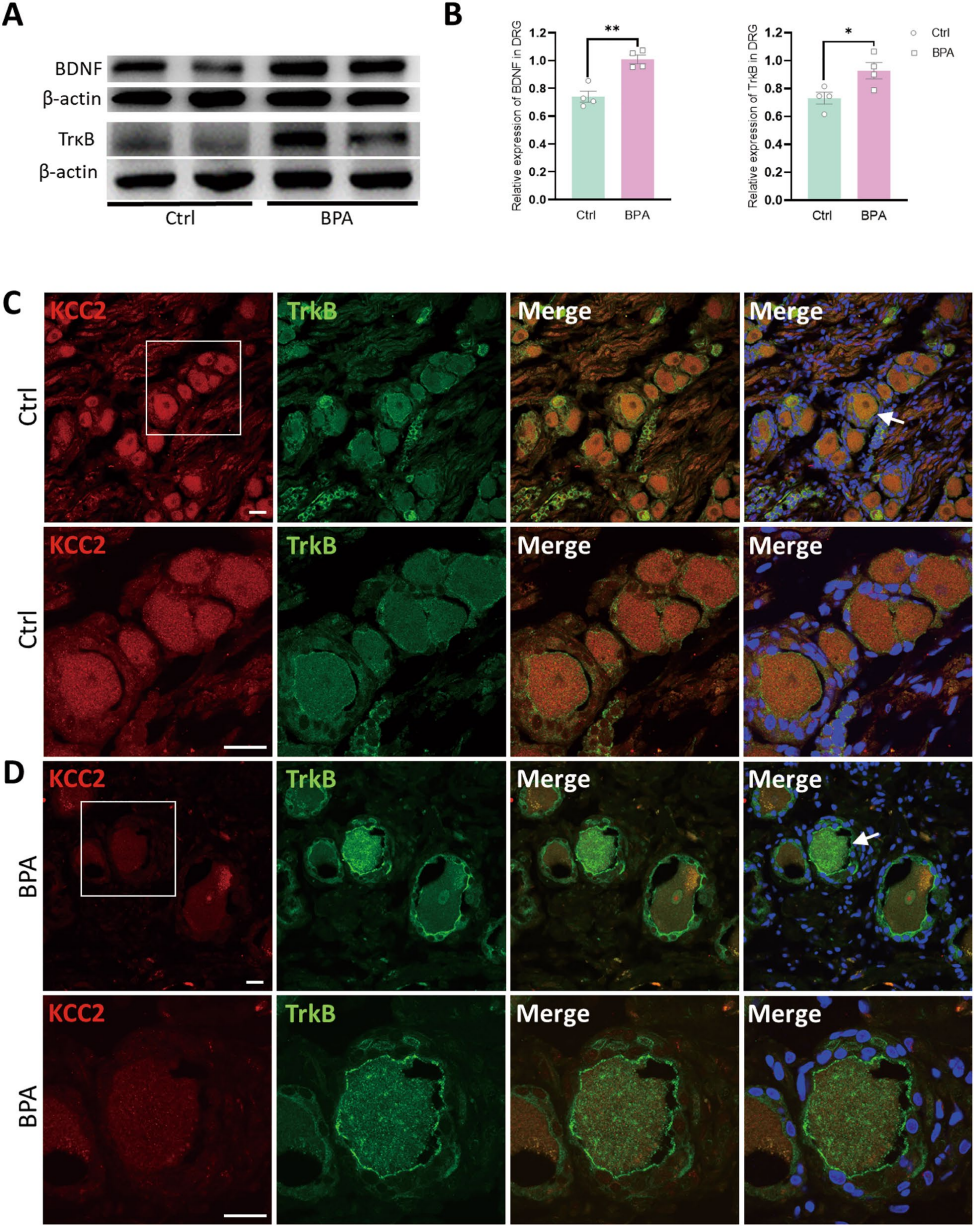
Expression of BDNF and TrκB shows similar tendency in the avulsed DRGs of clinical BPA patients. **A** Typical examples of western blots showing the expression of BDNF and TrκB in the DRG tissue of human control and BPA patients’ samples. **B** BPA increased the expression of BDNF and TrκB in the DRG of humans. Data are normalized to the housekeeping protein β-actin. **C-D** Representative confocal images of KCC2 (red) and TrκB (green) in the DRG tissue of human control and BPA patients’ samples using immunofluorescence labeling. The bottom rows are magnification images of interest area. Data are presented as the mean ± S.E.M. ^*^P < 0.05, ^**^P < 0.01, *vs* Ctrl. Scale bars, 20 μm. White arrows: co-localized neurons

As in clinic, patients with BPA often suffered by severe chronic pain of the affected extremity, and this kind of pain showed neuropathic characteristics including mechanical allodynia. The existing treatments show little analgesic effect on this kind of pain. To explore whether avulsed cervical nerve roots (including DRG soma) of BPA patients share similar molecular profile as BPA mice, we collected the avulsed nerve end of patients with chronic pain during surgery. A 31-year-old male patient was diagnosed with complete root avulsion of the brachial plexus according to a series of examinations including magnetic resonance imaging (MRI, SFig 7A). The onset of pain is early and becoming increasingly severe. The visual analogue scale (VAS) score of pain during first admission of this patient reached up to 8 (Table 1). The avulsed roots C6-T1 were collected (SFig 7B). The molecular changes of BDNF and TrκB showed similar tendency of change like BPA mice through western blot test (Fig. 8A, B). Immunofluorescence labeling showed increased fluorescence intensity of TrκB and decreased fluorescence intensity of KCC2 in DRG of BPA patients compared with normal ones (Fig. 8C, D). Interestingly, we can also observe that there’s little fluorescence of KCC2 in strongly TrκB-positive neurons in co-localization staining of DRG from BPA patients (Fig. 8D). In conclusion, similar changes in tendency of KCC2 and its upstream molecules both in mice and in humanity not only confirm the key role of KCC2 in BPA-induced NP, but also give us a possible molecular target for the treatment of this pain at the peripheral level in future clinical work.

## Discussion

The current study elucidates KCC2 can be a possible molecular therapeutic target of BPA-induced NP at the peripheral level, which is further confirmed through using tissues collected from BPA patients. This novel-designed BPA mice model showed persistent mechanical allodynia of the affected forepaw and single 7th root avulsion didn’t affect the motor function of the limb. The decreased expression of KCC2 in the affected DRG of BPA mice was confirmed through using both IHC at the translational level and FISH at transcriptional level. Specific genetic manipulation of KCC2 in peripheral DRG could reverse the hyperexcitation of DRG neurons and alleviate the pain state induced by BPA. Meanwhile, the calcium activity of the corresponding cervical SDH could be decreased synchronously. Possible molecular mechanism underlying this phenomenon was also explored. Nociceptor-localized KCC2 suppresses the release of its upstream BDNF from presynaptic terminals that innervated into superficial laminae of SDH, and this maybe the possible molecular basis of the analgesic function of it. Similar molecular changes of nerve roots from human present great translational potential of the current study to clinic.

Chronic pain is becoming a global burden worldwide that affects millions of people [34, 35]. Neuropathic pain (NP) often persists or manifests with recurrent painful states, with prevalence ranging from 6.9 to 10% of the general population according to epidemic data [5]. The chronicity characteristic of NP strongly suggests its complicated mechanisms during the occurrence and maintenance process. Diverse disorders including both function and molecular plasticity changes on the somatosensory pathway of pain transduction have been observed and reported [36, 37]. However, the mechanistic underpinnings of pain hypersensitivity and spontaneous pain induced by BPA are still poorly understood. Rodrigues-Filho et al. [38] reported the classical lower trunk BPA rat model and the detection of rats showed persistent mechanical and cold allodynia without affecting thermal hyperalgesia of both ipsilateral and contralateral hindpaws, and similar results were also observed on mice model reported by Quintão and his collegues [39]. Following BPA related research focused on exploring new model, pain mechanisms and motor neuron regeneration are gradually started, referring to narrative review by Xian H et al. [4]. However, an unsolved problem in terms of pain behavioral tests is all the behavioral tests are performed on other extremities except the avulsed one. Previous studies have proved glial cells were activated both in injured and uninjured level of cervical spinal cord [40], and Hou et al. [41] reported astrocytes activated on the avulsion side and microglia activated on bilateral side of cervical spinal cord under BPA-induced pain state. Interestingly, glial cells activation at higher C3-C4 level of dorsal horn in lower trunk C8-T1 of BPA may also play an important role in facilitating pain transmission [42]. These may be the reason why previous BPA models show mechanical pain on other paws except the injured paw. Multiple trunks avulsion of brachial plexus can lead to paralysis of the affected forelimb, which is unable to conduct pain behavior tests on the injured paw directly. Whether this indirect pain response detection can represent the real pain state of BPA models is still ambiguous. This novel designed single 7th root avulsion model we used in this study showed no impairment of motor function of the affected forepaw, which laid a good foundation of the following pain behavioral tests. Persistent mechanical allodynia through direct detection of the affected forepaw after BPA could be treated as a reliable parameter of pain state of BPA mice. Similar decreased mechanical threshold of the ipsilateral hindpaw could be considered as the result of central sensitization in spinal cord, which is in line with the previous reports [4]. While, whether top-down crosstalk occurred between cervical level and lumbar level in spinal cord under BPA-induced pain state is still unknown. Another problem is that the existing investigations are scattered, and there’s still a lack of in-depth study relating the integrality of pain transduction on the somatosensory pathway, especially on the level of crosstalk between peripheral and central levels.

Central sensitization is assumed to have close relationship with chronic pain. The peripheral nerve ending innervates into the superficial laminae of SDH where synaptic transmission first takes place, and the nociceptive signals from afferent fibers are enhanced during this process under pathophysiological conditions [43]. The increased release of neuroactive factors from nociceptive primary afferent terminals after nerve injury, including BDNF, was proven to produce and lead to pain hypersensitivity [33, 44]. The pivotal phenomenon of synchronized hyperexcitation activity at the level of both peripheral and central has recently been recognized in the pathophysiological process of NP [45, 46], while, investigation relating the underlying molecular basis remains sparse. Pain exists in other parts of the body except the injured area is considered as the external representation of central sensitization. Through using whole-cell patch‑clamp recording and spinal cord fiber photometry, the synchronized hyperexcitation activity of DRG (the peripheral level) and SDH (the central level) was detected under BPA-induced pain state. This could be the functional mechanism for that BPA mice showed pain behavior not only on the affected paw, but also on other paws. Thus, to further explore the underlying molecular mechanism, we focused on peripheral localized KCC2. Overexpression of KCC2 in DRG could reverse the decreased mechanical allodynia induced by BPA of both ipsilateral forepaw (the peripheral level) and hindpaw (the central level), and agonist of KCC2 can lower the hyperexcitation of DRG neurons and relive pain at the same time. Interestingly, level of calcium activity of SDH could also be reduced through peripheral manipulation of KCC2. These results provide the evidence on the crosstalk between PNS and CNS after BPA-induced pain sensitization, and the central sensitization could be regulated through the peripheral method by targeting KCC2 in DRG neurons.

The movement of Cl^‐^ is crucial to neuronal Cl^‐^ homoeostasis and plays an essential role in regulating neuronal excitability [16]. KCC2, encoded by the *Slc12a5* gene, as a member of the cation‐chloride cotransporters (CCCs) family, is a membrane protein that can actively extrude intracellular Cl^‐^, which is important for consequently functional GABAergic inhibition. While, inhibition of GABAergic neurons has been well studied at the CNS level, more and more GABA_A_ receptors have also been identified at the PNS level. It is reported that α2, β3, β2 and γ subunits of GABA_A_ receptor expressed in DRG soma and nerve fiber ends, and the plasticity change expression of them under pain state showed their different roles in pain modulation [44]. Several related functions of KCC2 in the CNS have been well studied, nevertheless, whether peripheral level expressing KCC2 remains controversial [16, 47, 48]. Funk K et al. [49] confirmed the expression of KCC2 declined in DRG neurons and its role in the generation of hyperalgesia, while other studies found no evidence of KCC2 expression in PNS [31, 50]. We conducted IHC and FISH technology to test the expression of KCC2 in DRG of BPA mice, and the results showed the fluorescence intensity decreased. Quantitative analysis using western blot showed the same decreased expression profile of KCC2 in DRG under BPA-induced pain state. These evidence confirmed the expression of KCC2 in DRG, and the function based research above proved its analgesic role at the peripheral level. These findings also provide evidence that GABA_A_ receptor can function normally at the peripheral level.

BDNF, as a member of neurotrophin family of neurotrophic factors, is known for its effects on neuronal survival and growth, also playing crucial role in synaptic development and plasticity [51, 52]. BDNF protein is synthesized in cell bodies of neurons and glial cells, which is proven to participate in pathophysiological process like NP [53, 54]. At the level of CNS, overexpression of BDNF after injury can lead to downregulation of the downstream KCC2 through its receptor TrκB, which is critical in regulating excitation homeostasis condition of neurons in the superficial lamina of SDH and spinal projection neurons, and this downregulation can further impair the inhibition effect of GABAergic neurons, thus initiates pain [36, 55]. This mechanism of disinhibition effect mediated by BDNF-TrκB-KCC2 signaling is also proved in higher level of CNS during chronic pain [56]. Accumulating evidence shows persistent activation of peripheral nociceptors following nerve injury/inflammation could enhance the presynaptic input from periphery to postsynaptic dorsal horn [57]. Numerous neurotransmitters released from nociceptive terminals participate in this pathological process [12, 33, 58]. Our study identified BPA-induced the overexpression of BDNF in DRG, and the hyperexcitation state of neurons can increase excitatory evoked BDNF release from nociceptive terminals to the superficial lamina of the dorsal horn. Overexpression of KCC2 in DRG could in turn reduce the synthesis and release of BDNF and achieve pain relief. These evidence strongly suggest that peripheral KCC2 can suppress BPA-induced neuropathic pain and related central sensitization through its upstream BDNF signaling in reverse. Surprisingly, the expression tendency of KCC2 and its upstream BDNF and TrκB in tissues derived from BPA patients is consistent with the changes in BPA mice, and the KCC2 positive neurons have a close colocalization relationship with TrκB also hinting their close interaction possibility. This provides a robust evidence of clinical transformation in the future.

## Conclusions

In sum, our study showed the possibility of manipulating central sensitization through peripheral method, which could avoid numerous side effects of CNS medication delivery. This molecular based therapeutic method also points to a novel therapeutic strategy with promising prospect of clinical translation. On considering the possible molecular mechanism mediated by peripheral KCC2, we proved DRG localized KCC2 participated in feedback down regulation of its upstream BDNF at the DRG to SDH loop level, which provides a theoretical basis for the future accurate treatment of this kind of pain. Yet, unraveling the complex mechanisms of pain is still a long way to go, and investigations based on human tissues will do accelerate the pace for future clinical applications.

## Supplementary Information

Additional file 1.

Additional file 2.

Additional file 3.

Additional file 4.

Additional file 5.

Additional file 6.

Additional file 7.

## Data Availability

The original contributions presented in the study are included in the article, and further inquiries can be directed to the corresponding authors.

## Abbreviations

BPA: Brachial plexus avulsion
NP: Neuropathic pain
KCC2: K^+^-Cl^−^ cotransporter 2
BDNF: Brain derived neurotrophic factor
DRG: Dorsal root ganglion
PNS: Peripheral nervous system
CNS: Central nervous system
SDH: Spinal dorsal horn
mPFC: Medial prefrontal cortex
TrkB: Tropomyosin-related kinase B
IHC: Immunohistochemistry
FISH: Fluorescence in situ hybridization
DEPC-PBS: Diethylpyrocarbonate-treated phosphate-buffered saline
RMP: Resting membrane potential
AP: Action potential
AP AHP: AP after hyperpolarization potential
