# Supplementary figures and images for: Nociceptor-localized KCC2 suppresses brachial plexus avulsion-induced neuropathic pain and related central sensitization
Source: Cell Biosci. 2025 Jan 31;15:12. doi: 10.1186/s13578-025-01354-5 (PMC11786554; doi:10.1186/s13578-025-01354-5)

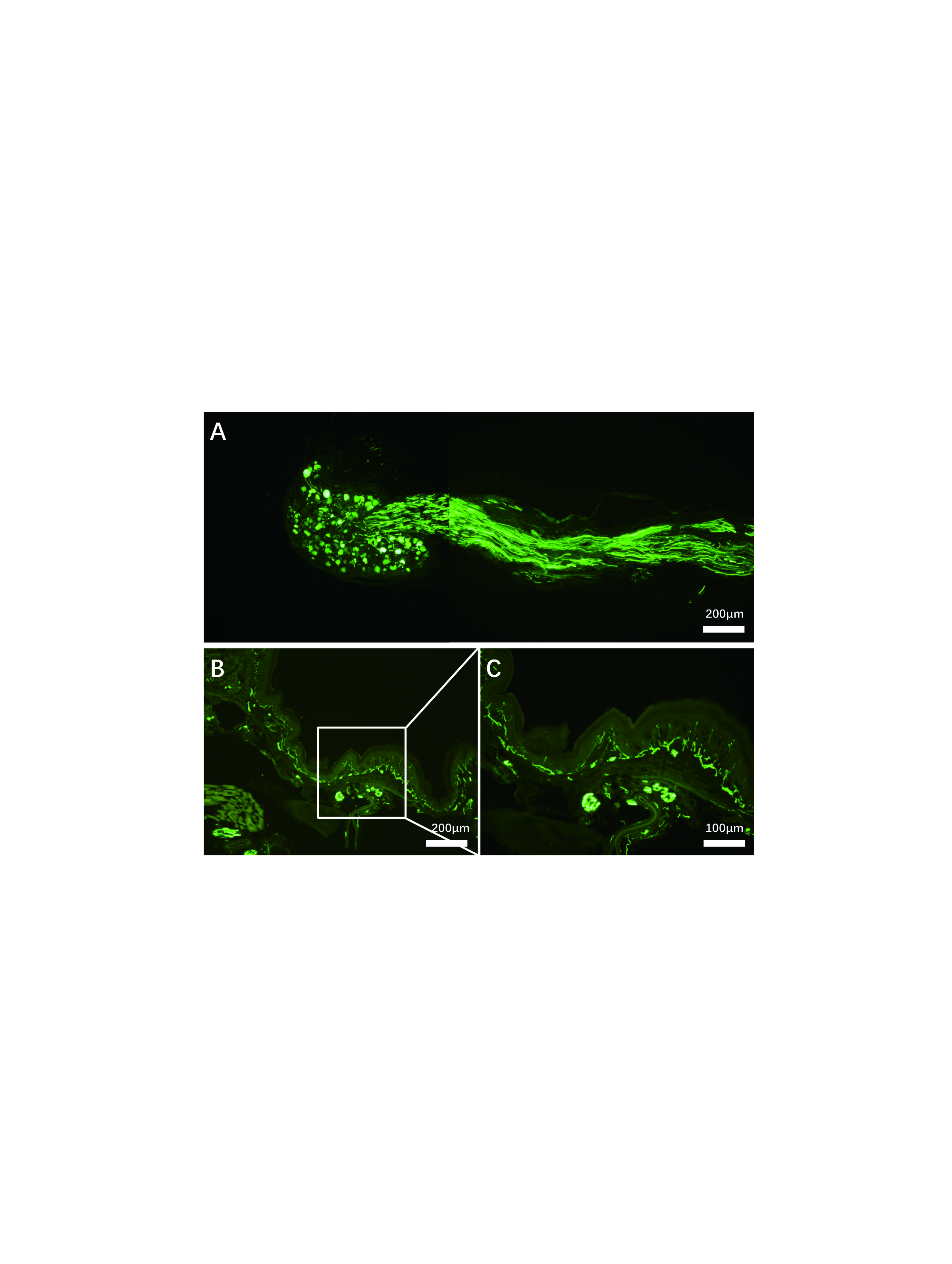

Supplement: Supplementary file 1 — Additional file 1. [file 13578_2025_1354_MOESM1_ESM.tif]

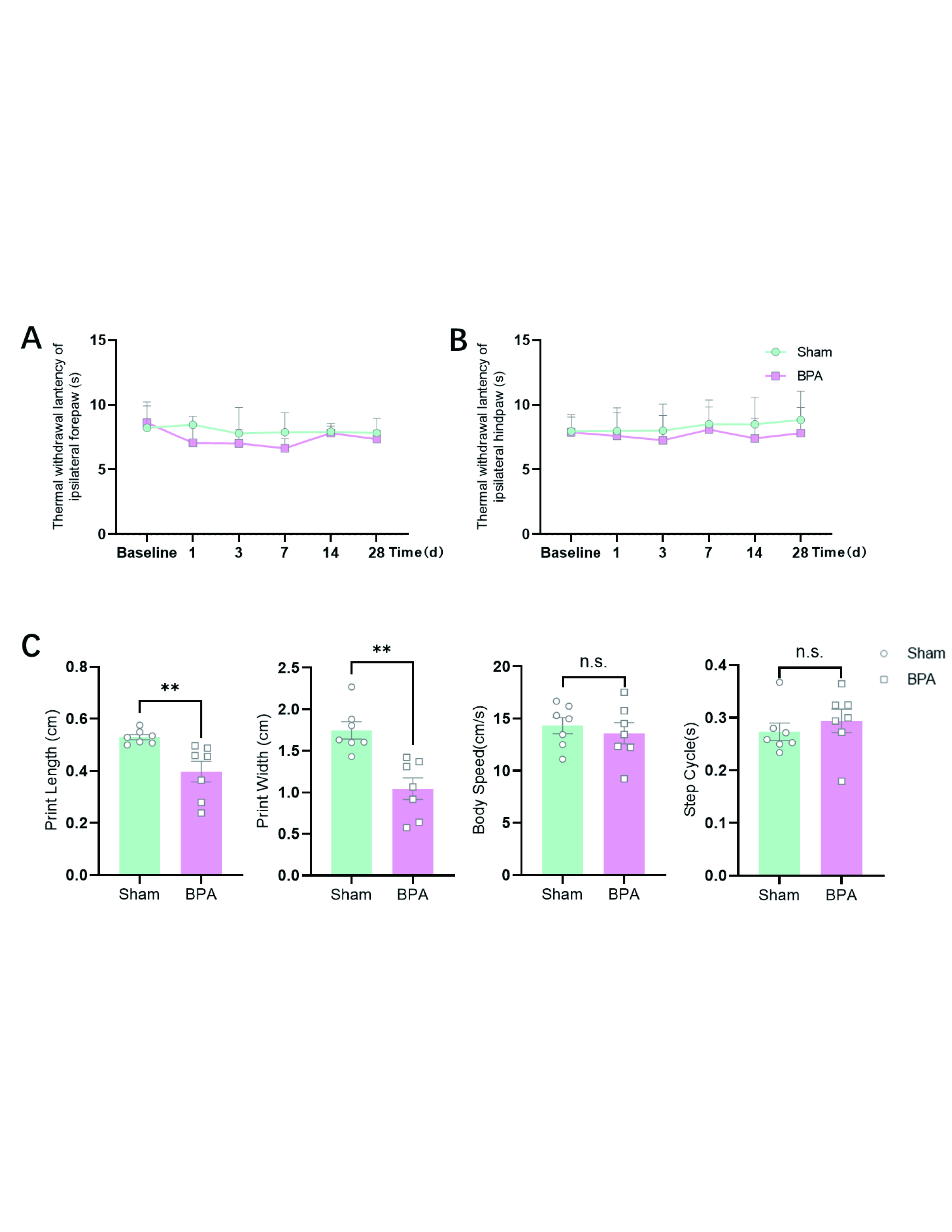

Supplement: Supplementary file 2 — Additional file 2. [file 13578_2025_1354_MOESM2_ESM.tif]

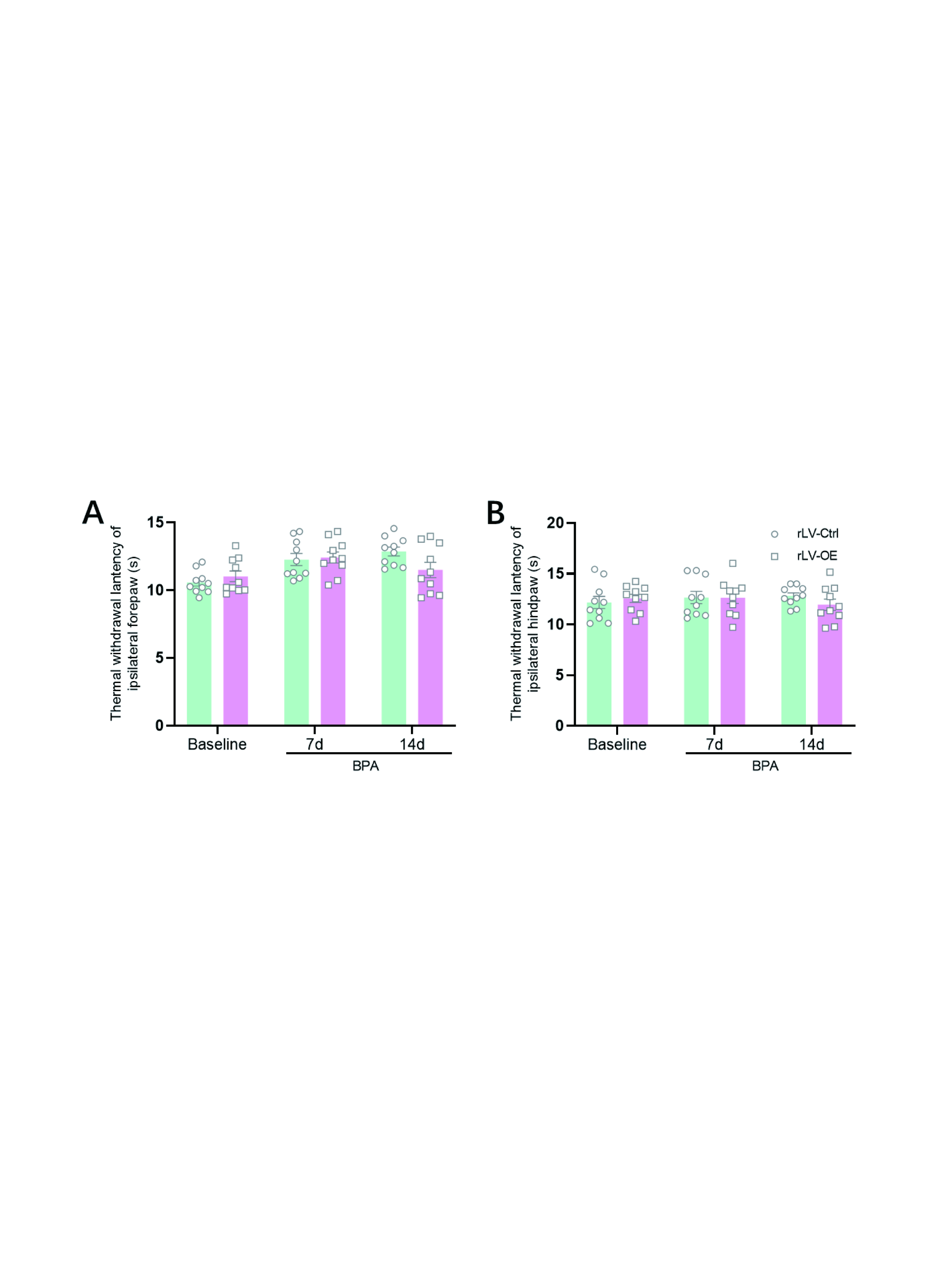

Supplement: Supplementary file 3 — Additional file 3. [file 13578_2025_1354_MOESM3_ESM.tif]

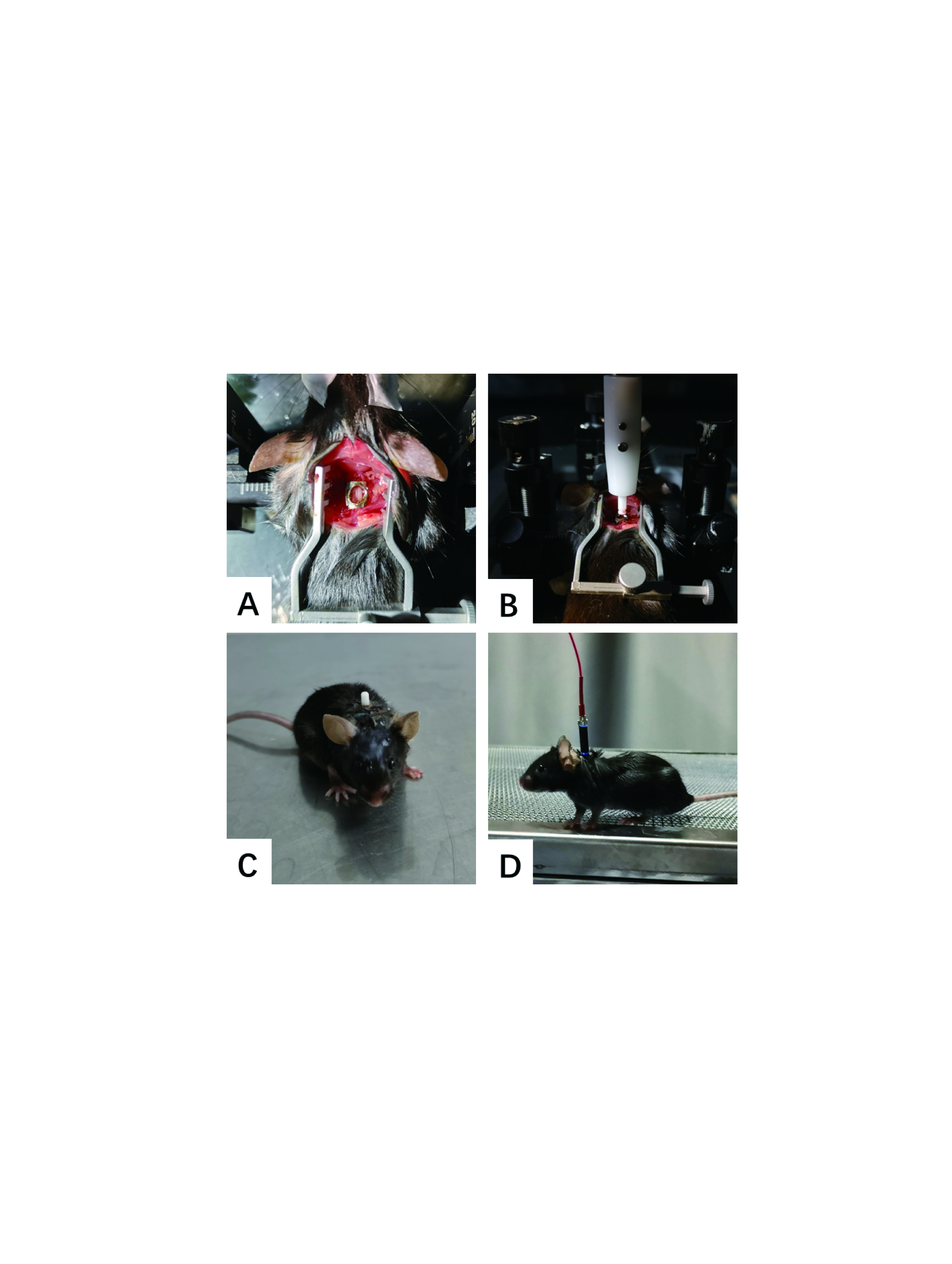

Supplement: Supplementary file 4 — Additional file 4. [file 13578_2025_1354_MOESM4_ESM.tif]

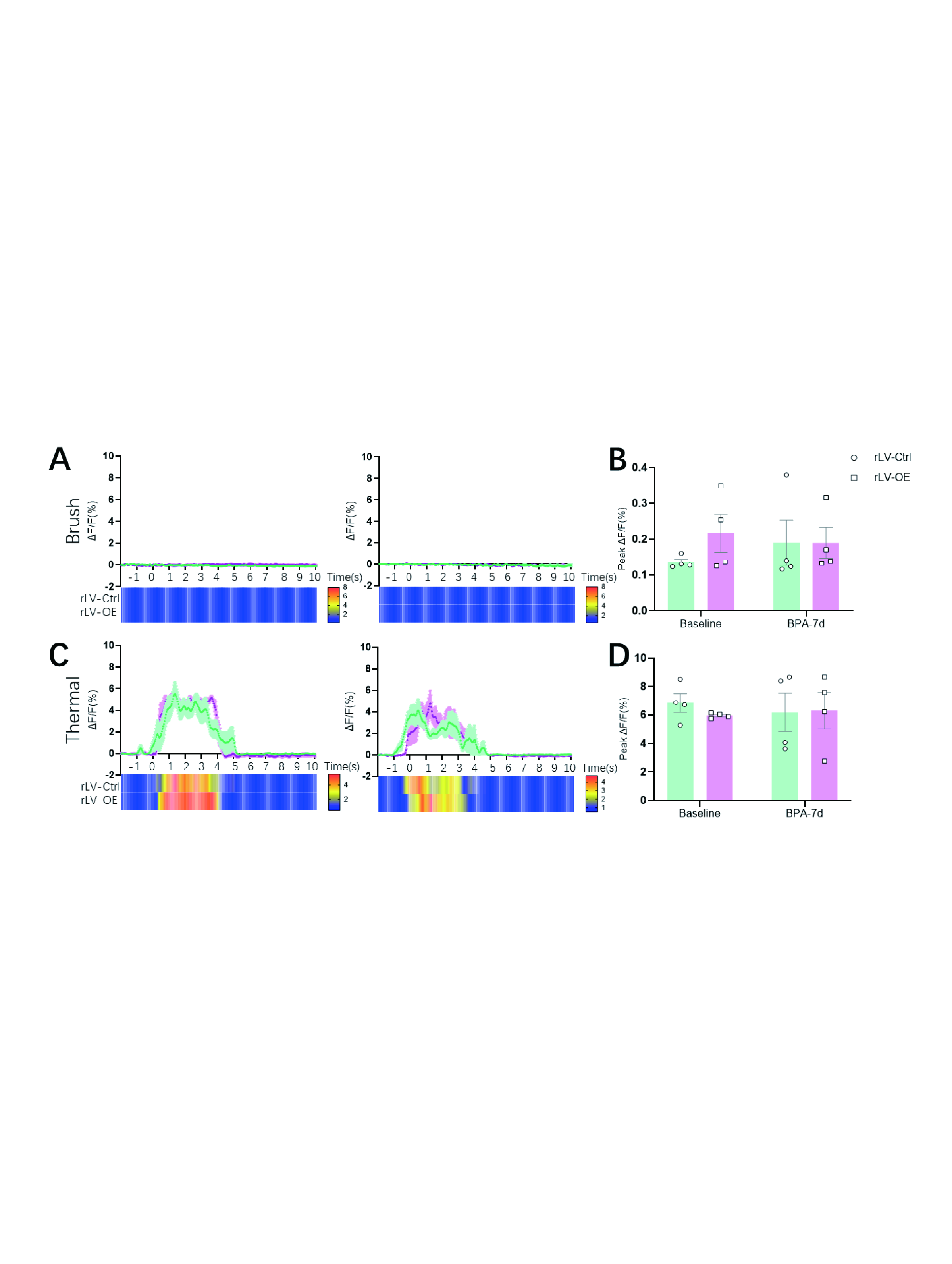

Supplement: Supplementary file 5 — Additional file 5. [file 13578_2025_1354_MOESM5_ESM.tif]

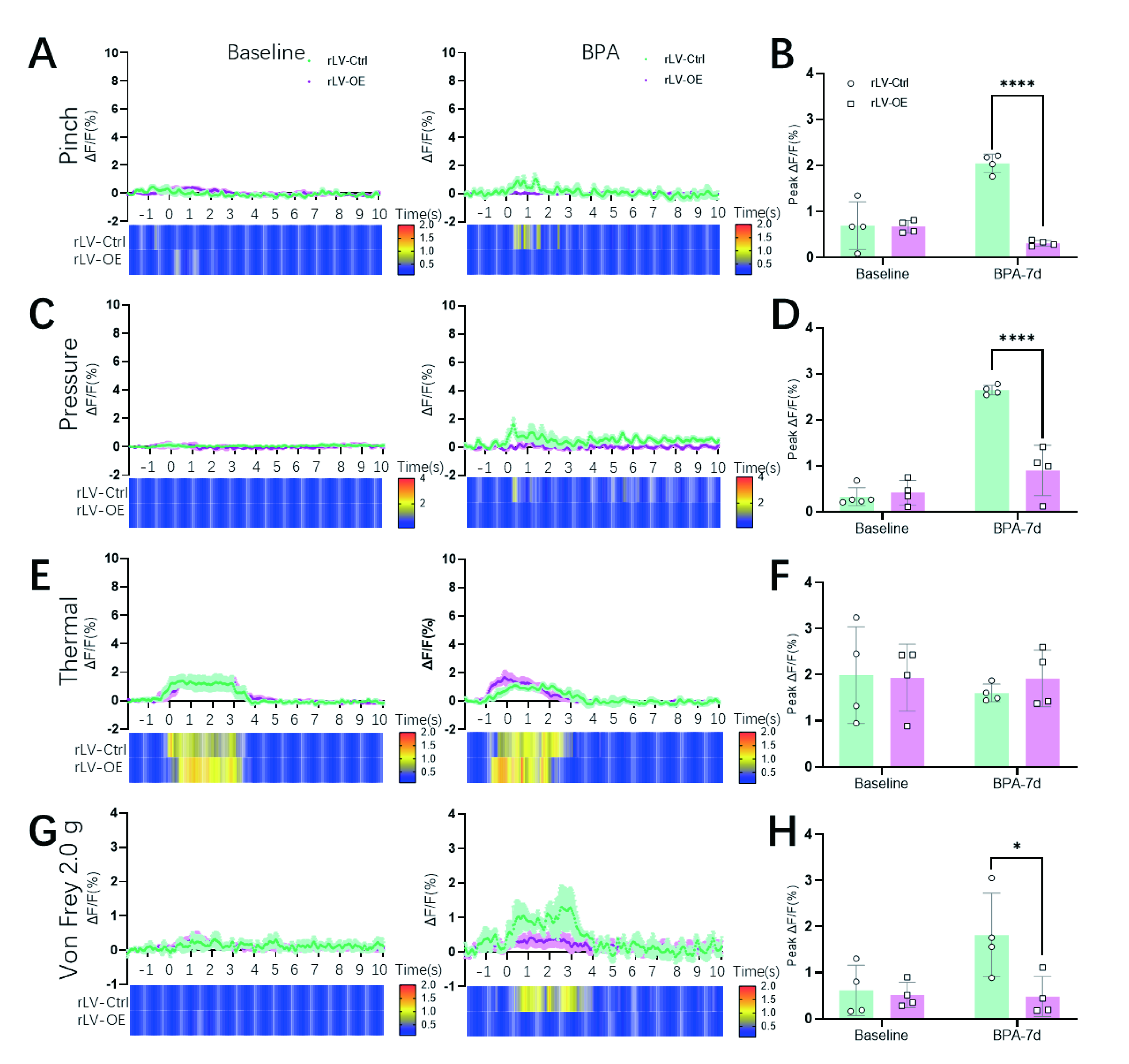

Supplement: Supplementary file 6 — Additional file 6. [file 13578_2025_1354_MOESM6_ESM.tif]

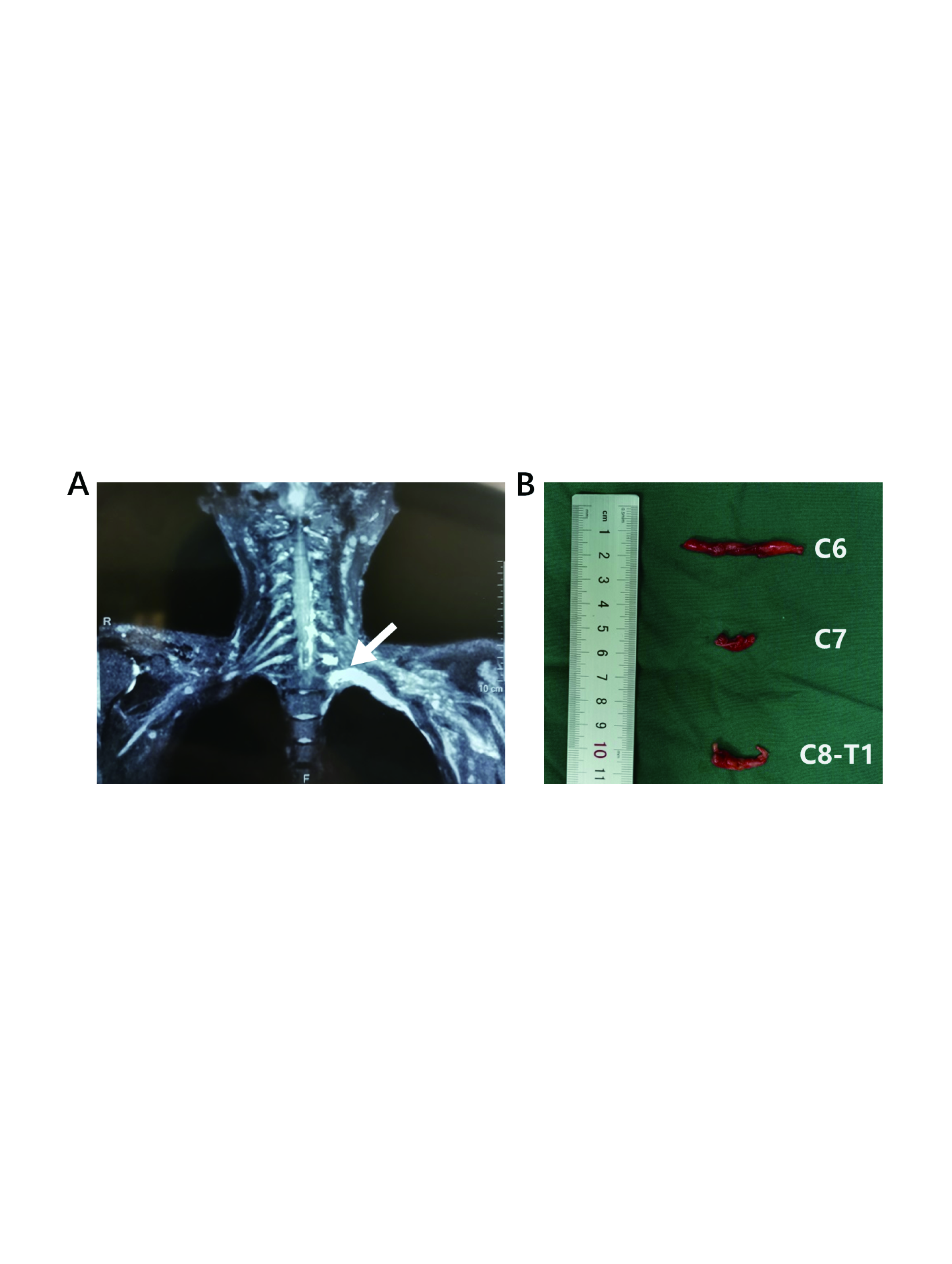

Supplement: Supplementary file 7 — Additional file 7. [file 13578_2025_1354_MOESM7_ESM.tif]
